# Supplementary material for: Asgard archaea modulate potential methanogenesis substrates in wetland soil
Source: Nat Commun. 2024 Jul 31;15:6384. doi: 10.1038/s41467-024-49872-z (PMC11291895; doi:10.1038/s41467-024-49872-z)
Supplement: Supplementary file 1 — Supplementary Information [file 41467_2024_49872_MOESM1_ESM.pdf]

# SUPPLEMENTARY INFORMATION

## Asgard Archaea Modulate Potential Methanogenesis Substrates in Wetland Soil

Valentin-Alvarado et al.

**Table of content** **Page number**

| Table of content | Page number |
| --- | --- |
| Supplementary Text | 2-5 |
| Supplementary Figures | 6-30 |

## Supplementary Text

**Modified EMP Pathway:**
Atabeyarchaeia and Freyarchaeia use the Embden-Meyerhof-Parnas (EMP) glycolytic pathway, producing ATP through fermentation of acetyl-CoA to acetate. The entry point into the pathway is F6P for the Atabeyarchaeia genomes and glucose for Freyarchaeia. In addition to the different entry points, they utilize different enzymes to produce pyruvate. Atabeyarchaeia encodes the oxygen-sensitive reversible enzyme, pyruvate phosphate dikinase (ppdK; IPR010121); whereas Freya encodes unidirectional pyruvate water dikinase/phosphoenolpyruvate synthase (pps; IPR006319) and pyruvate kinase (pk; IPR001697), producing phosphoenolpyruvate and pyruvate, respectively. The pyruvate phosphate dikinase found in Atabeya is proposed in archaea to be an ancestral version of components of the glycolytic pathway in anaerobic eukaryotes [107], **Supplementary Fig. 15**). Freyarchaeia encoding a complete EMP Pathway supports previous descriptions of Freyarchaeia MAGs from sediments and other Asgard archaea, including Lokiarchaeia, Hermodarchaeia, Thorarchaeia, Odinarchaeia, Sifarchaeia, and Heimdallarchaeia[10,12]. Less common among the described Asgard is starting at F6P, lacking the traditional enzymes for the degradation of glucose at the start of the EMP Pathway. In this capacity, Atabeyarchaeia more closely resembles the pathway's description for Helarchaeales, an order of Lokiarchaeia[7]. Helarchaeales have not been shown to rely on simple sugars, but instead, utilizes alkanes as an alternative carbon source, connecting glycolysis to a partial TCA cycle. From the two complete Atabeyarchaeia genomes, we were able to map transcripts from G6P to 2PG and PEP to acetate. Atabeyarchaeia transcripts supported metagenomic data, connecting the TCA cycle (oxaloacetate) to EMP Pathway (PEP) as both genomes transcribed phosphoenolpyruvate carboxykinase. Freya transcribed 3PG to 2PG and PEP to acetate. Like Atabeyarchaeia, Freyarchaeia transcribed enzymes connecting malate and oxaloacetate in the TCA cycle to EMP and a portion of RuMP/NO-PPP, connecting H6P to F6P.

**Partial TCA Cycle:** Atabeyarchaeia and Freyarchaeia resemble previous descriptions of Asgardarchaeota, encoding only partial TCA cycle, connecting this pathway to the EMP Pathway and amino acid degradation (i.e., glutamate). The main difference between lineages in this pathway is that Atabeyarchaeia encodes fumarate hydratase, catalyzing the reversible reaction of fumarate to malate. The only portion of the TCA cycle transcribed in Atabeyarchaeia and Freyarchaeia is 2-oxoglutarate/2-oxoacid ferredoxin oxidoreductase, producing reducing power in NADH.

**Hydroxyglutarate Pathway:** Freyarchaeia and Atabeyarchaeia appear to ferment amino acids via the partial hydroxyglutarate pathway. Of the three main hydroxyglutarate pathway steps, both lineages encode the first reversible NADH-dependent reduction of 2-oxoglutarate to 2-hydroxyglutarate (step 22 in Fig. 2), connecting amino acid fermentation to the partial TCA cycle and therefore production of ATP and NADPH or NADH reducing equivalents. Only Freyarchaeia has the enzymes for the dehydration of hydroxyglutaryl-CoA to glutaconyl-CoA (HgdAB) and both groups lack the enzyme necessary to decarboxylate glutaconyl-CoA to crotonyl-CoA [108]. The encoded hydroxyglutarate pathway enzymes (HgdAB, GdhA, PHGD, GctAB) may produce H+, ammonium, acetate and hydroxyglutaryl-CoA. Assuming there is a mechanism to convert glutaconyl-CoA to crotonyl-CoA, fermentation of crotonate would produce NADH, $FADH_2$ and acetyl CoA. This pathway links amino acid fermentation to central metabolism and production of ATP and is consistent with descriptions of anaerobic amino-acid degradation in the last Asgard common ancestor [4,9].

**Reductive Glycine Pathway (rGlyP):** We identified genes predicted to be involved in the anaerobic oxidation of glycerol and the glycine cleavage system (GCS), which is part of the

reductive glycine pathway (rGlyP). All three genomes encode glycerol-3-phosphate dehydrogenase complex (*glpABC*), a putative glycerol kinase (*glpK*), and glycerol dehydrogenase (*gldA*), as well as, P, T and H proteins of GCS. Sequences from Atabeyarchaeia and Freyarchaeia are phylogenetically distinct from characterized glycerol-3-phosphate dehydrogenase subunit A (*glpA*), however Freyarchaeia clusters with Halobacteriales known to metabolize glycerol[109] (Supplementary Table 7). Reverse GlyP (rGlyP) has been identified in other archaea but this pathway in Atabeyarchaeia and Freyarchaeia is unique in that it resembles *E. coli*, lacking the L-protein (dihydrolipoyl dehydrogenase), reducing NAD+ to NADH [47,110]. Despite the discussion of glycine metabolism in archaea little is known about the the rGlyP in Asgard archaea [9,11]. Yet the ancestral reconstructions suggest the glycine cleavage system was present in the Asgard archaeal ancestor, which supports the suggested deeper phylogenetic branching of these two soil Asgard clades [4].

**Hydrogenases:** Atabeyarchaeia and Freyarchaeia generate energy through various NiFe hydrogenases, incomplete electron transport chain, and glycerol respiration. Both clades harbor Group 4g and Group 3c [NiFe]-hydrogenases. Membrane-bound Group 4 [NiFe] hydrogenase [111] has previously only been reported in Hermod-, Heimdall- and Odinarchaeia. As Atabeyarchaeia and Freyarchaeia lack methyl coenzyme-M (*mcrABC)* genes, HdrA2B2C2 may function bidirectionally, facilitating both hydrogen oxidation and the formation of a bifurcating complex, coupling the presence of Group 3c [NiFe]-hydrogenase (*MvhADG*) genes. Previous descriptions of Asgard and TACK lineages have suggested Group 3c [NiFe] hydrogenase: heterodisulfide reductase-linked hydrogenases are involved in energy conservation through electron bifurcation[1,48]. Similar to other TACK lineages, both Atabeya genomes have Group 3b [NiFe]-(sulf)hydrogenase, coupling oxidation of NADPH to fermentative production of $H_2$ or sulfhydrogenase activity, reducing elemental sulfur to hydrogen sulfide previously reported in *Pyrococcus furiosus*[112]. The addition of Group 3b [NiFe]-hydrogenase (*HydABCD*) in Atabeyarchaeaia enables additional hydrogen formation, which is absent in Freyarchaeia [27,113,114]. The presence of [NiFe]-hydrogenase Group 3b and 3c indicates that these archaea potentially use $H_2$ as an electron donor, or more likely they generate fermentation capacity, as suggested by others[35,115]. A membrane-bound succinate dehydrogenase complex and an A-type ATP synthase complex are encoded in Freyarchaeia and Atabeyarchaeia. Both groups are capable of transferring electrons from glycerol-3-phosphate generated from the reduction of menaquinol to reduce fumarate to succinate as the final electron acceptor in the cell[116].

**Alcohol Dehydrogenases and Butanol Oxidation:** Atabeyarchaeia and Freyarchaeia complete genomes encode iron-containing alcohol dehydrogenase (Fe-ADH)-like enzymes, which likely participates in both fermentation and production of acetyl-CoA. This capacity is also predicted in Hel-, Njord-, and Lokiarchaeales, as supported by phylogenetic analysis **(Supplementary Fig. 24**). In particular, the Freyarchaeia complete genomes contains a potential NADPH-dependent butanol dehydrogenase (BDH) part of the reversible butanol oxidation/pyruvate fermentation to butanol pathway, a prevalent fermentation product in both wetland sediments and terrestrial carbon cycling (**Supplementary Fig. 24**). Butanol can be used as a substrate for anaerobic methanogenesis, converting butanol into butyrate, acetate, and methane, sequentially.

**Putative Aerobic-like Carbon-Monoxide Dehydrogenase (CoxLMS):** We also identify putative genes for aerobic carbon-monoxide dehydrogenase (CoxLMS) and cofactors within Freyarchaeia. The putative CoxL forms a monophyletic group with other archaea, suggesting a potential capacity for carboxydotrophy or the utilization of alternative substrates in the presence of oxygen, such as aldehydes or purines, as a member of the aldehyde oxidase superfamily. (**Supplementary Fig. 17**)

Besides the bifunctional carbon monoxide dehydrogenase that is part of the WLP and generates acetyl-CoA, Freyarchaeia also encodes putative genes for aerobic carbon-monoxide dehydrogenase (CoxLMS) and cofactors which could oxidize CO as an additional electron donor, suggesting a potential capacity for carboxydotrophy. However, phylogenetic analysis shows that the CoxL from Freyarchaeia belongs to a monophyletic group and clusters with other uncharacterized archaeal CoxL and does not cluster with biochemically characterized form I CoxL suggesting the potential use of other substrates.

**Biosynthesis of Coenzymes and other metabolic precursors:** Other operons suggest that Atabeyarchaeia and Freyarchaeia can self-synthesize MoCF (Molybdenum Cofactor, Molybdopterin), a cofactor enabling the functionality of a wide spectrum of enzymes (i.e., xanthine dehydrogenase superfamily), generating NADH or NADPH. Both lineages also have the enzyme for the biosynthesis of coenzyme A (*pok*), a precursor in various metabolic processes, including the previously discussed TCA cycle and amino acid metabolism. Additionally, Freyarchaeia has enzymes for the biosynthesis of coenzymes B (*leuABCD*), M (*thrC*, *comABCDE*), and F420 (*cofCDEGH*) not encoded in Atabeyarchaeia. The Asgardarchaeota sister-lineage TACK has been shown to have similar enzymes for cofactor biosynthesis [48].

**Metatranscriptomics:** Transcriptomic data indicates *in situ* expression acetogenic and energy conservation pathways in both Atabeyarchaeia and Freyarchaeia complete genomes, including EMP glycolysis/gluconeogenesis (**Supplementary Fig. 15**), WLP (**Supplementary Fig. 11**), beta oxidation, formaldehyde oxidation, RuMP (**Supplementary Fig. 16)**, Group 3c [NiFe] Hydrogenase, ATP synthase (**Fig. 2**, step 82), NADH-quinone oxidoreductase (**Fig. 2**, step 85), and molybdopterin biosynthesis (**Supplementary Data 8)**. We also identified transcription for environmental and stress response, including nickel, arsenite, magnesium, iron, and copper transporter, heat shock proteins, catalase, and superoxide dismutase.

Both Atabeya-1 and Atabeya-2 had more than 10 copies of sugar and amino acid transporters, and GT CAZYmes for glycosylation activities, providing the substrates for the fermentation of sugars and amino acids. In Atabeya-1, we identified another transcribed (>10 transcript) gene (*pckA*), potentially replenishing the phosphoenolpyruvate (PEP) pool for glycolysis/gluconeogenesis. The eukaryotic signature protein, Lokiactin, was expressed in Atabeyarchaeia, being the most expressed gene in Atabeya-1. Atabeya-2 had more than 10 copies for the Group 3b [NiFe] hydrogenase components not found in Freyarchaeia, *mtrA*, *aor,* both a peptidase inhibitor (I87) and an asgparagine peptide lyase (N11), and amino acid metabolism genes (*trpB, kce, iorA*) (**Supplementary Data 8**).

In Freyarchaeaia, we identified more than 10 transcript copies of beta-glucosidases, cytoplasmic/cytoplasmic membrane CAZYmes (AA0, CBM50, GH1, GH3, GT3, GT7, and GT66), asparagine peptide lyase (N11), beta oxidation, *aor*, and an intermediate step of the WLP H4MPT branch (*mer*). Environmental stress responses were among the most transcribed, especially the catalase-peroxidases not found in Atabeyarchaeia, potentially detoxifying hydrogen peroxide with methanol to produce formaldehyde, superoxide dismutase, small heat shock proteins, and copper detoxification transport **(Supplementary Data 8)**.

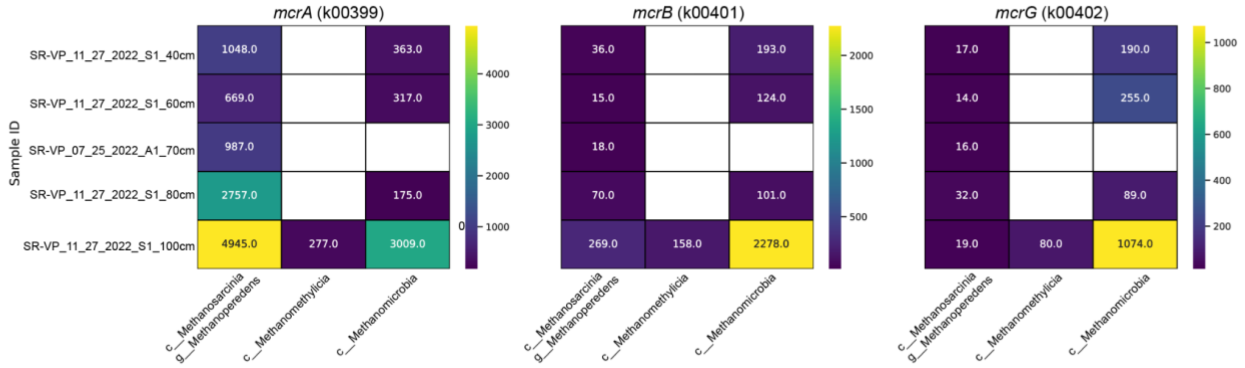


**Supplementary Figure 1** Expression profiles of MCR complex genes in 3 methane metabolizing archaea from the study site. Heatmaps illustrating average RNA counts for genes K00399 (alpha subunit), K00401 (beta subunit), and K00402 (gamma subunit) of the methyl-coenzyme M reductase complex in methanogens and methane-oxidizing archaea. Expression levels are shown for five samples arranged by increasing depth: 40cm, 60cm, 70cm, 80cm, and 100cm. Color intensity represents expression levels, with warmer colors denoting higher expression. These samples were regenerated using pooled replicates so is not direct evidence of varioatins across samples rather is to illustrate the expression levels of the archaea present in the samples.

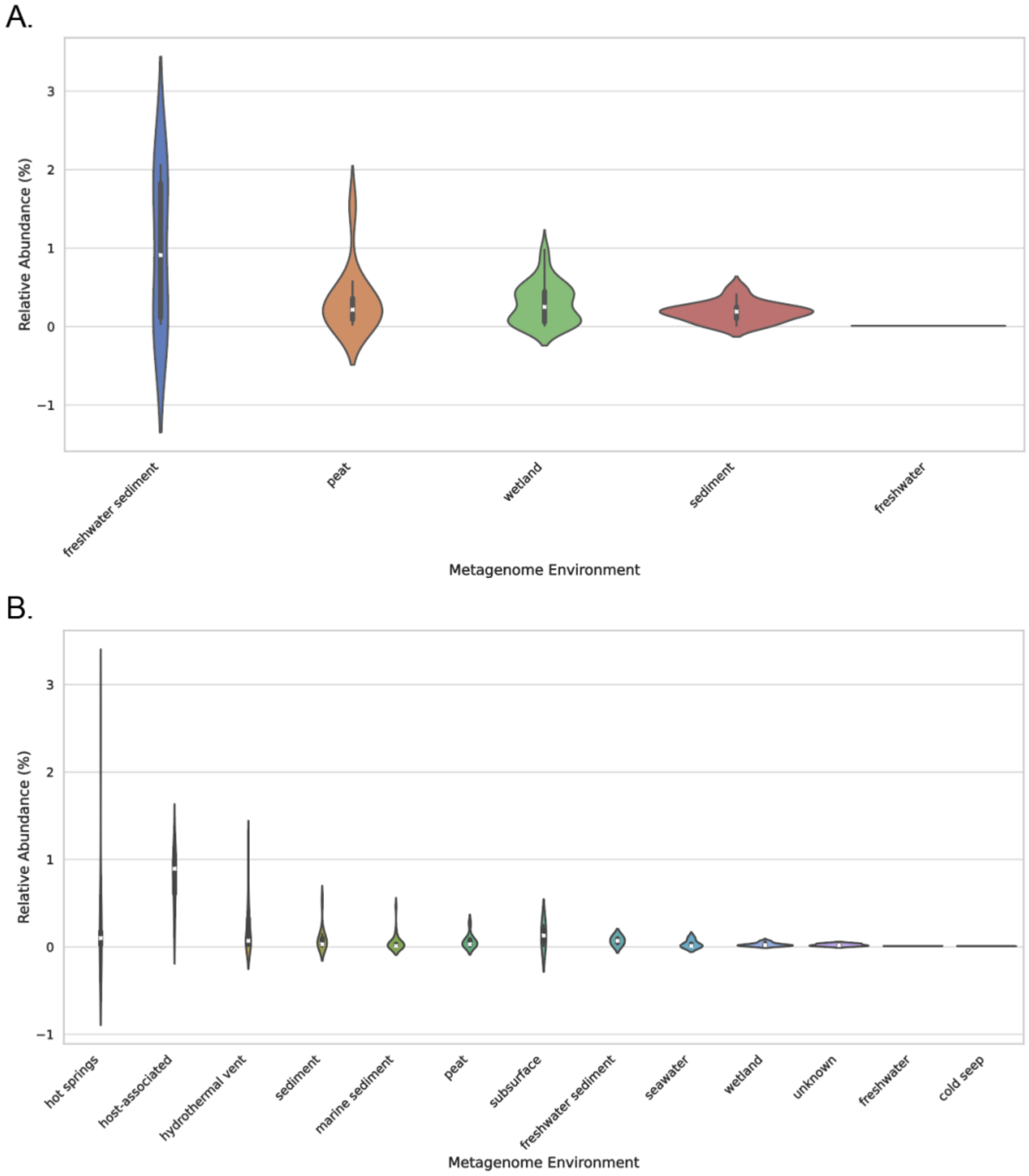


**Supplementary Figure 2** Distribution of Atabeyarchaeia (a) and Freyarchaeia (b) in publicly available metagenomes. Violin plots illustrate the relative abundance of Atabeyarchaeia and Freyarchaeia across various metagenome environments. The x-axis denotes the type of metagenomic environment sampled, and the y-axis represents the relative abundance percentage. Data were filtered to exclude categories with a relative abundance below 0.01%. Each plot highlights the variability and density of abundance in different environments, demonstrating the ecological distribution of these organisms within the metagenomic data.

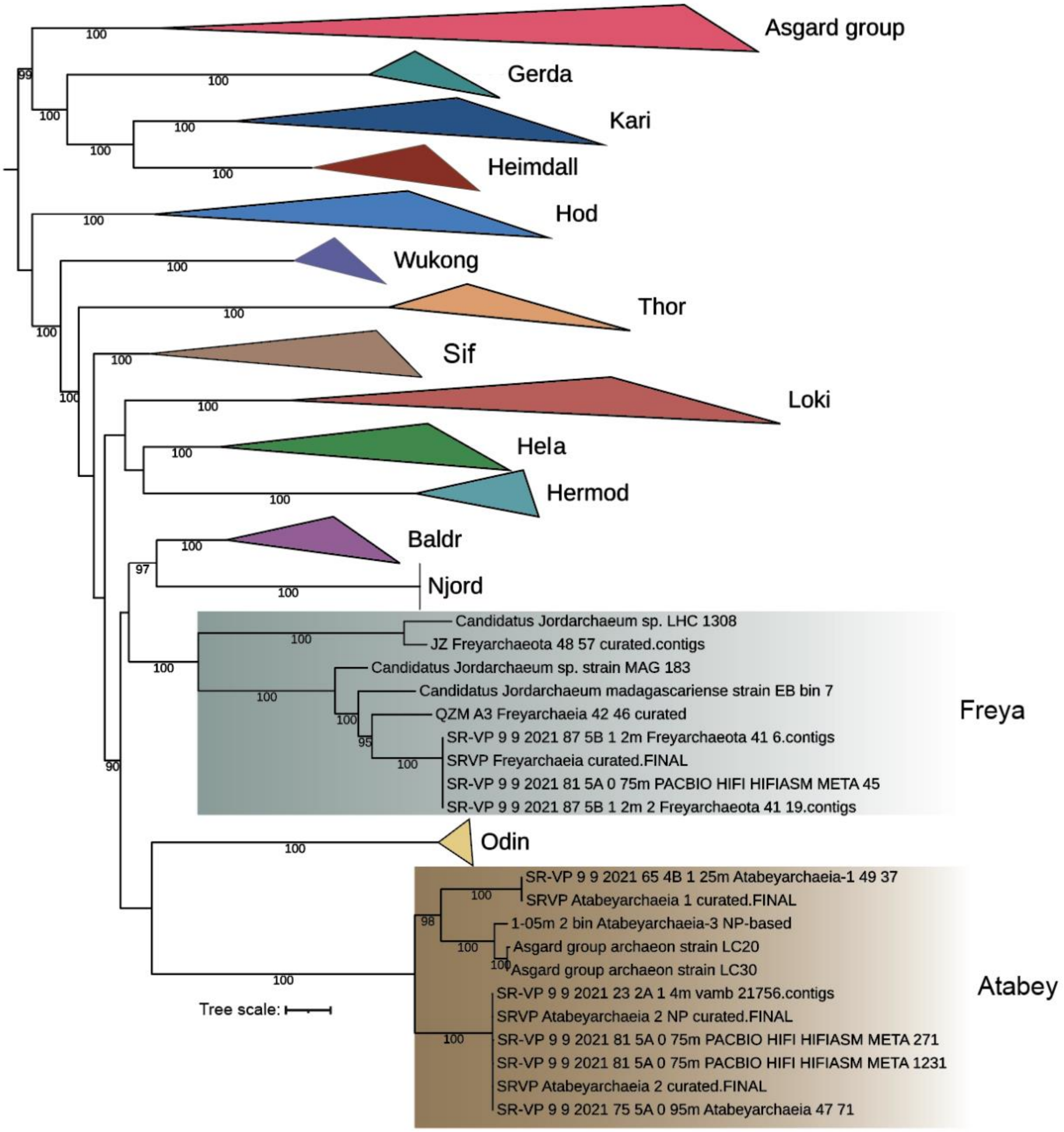


**Supplementary Figure 3** Maximum-likelihood tree, inferred with IQtree and the best-fit LG+C20+F+G model, using a concatenated set of RP15. Ultrafast bootstrap support values of >90 are shown.

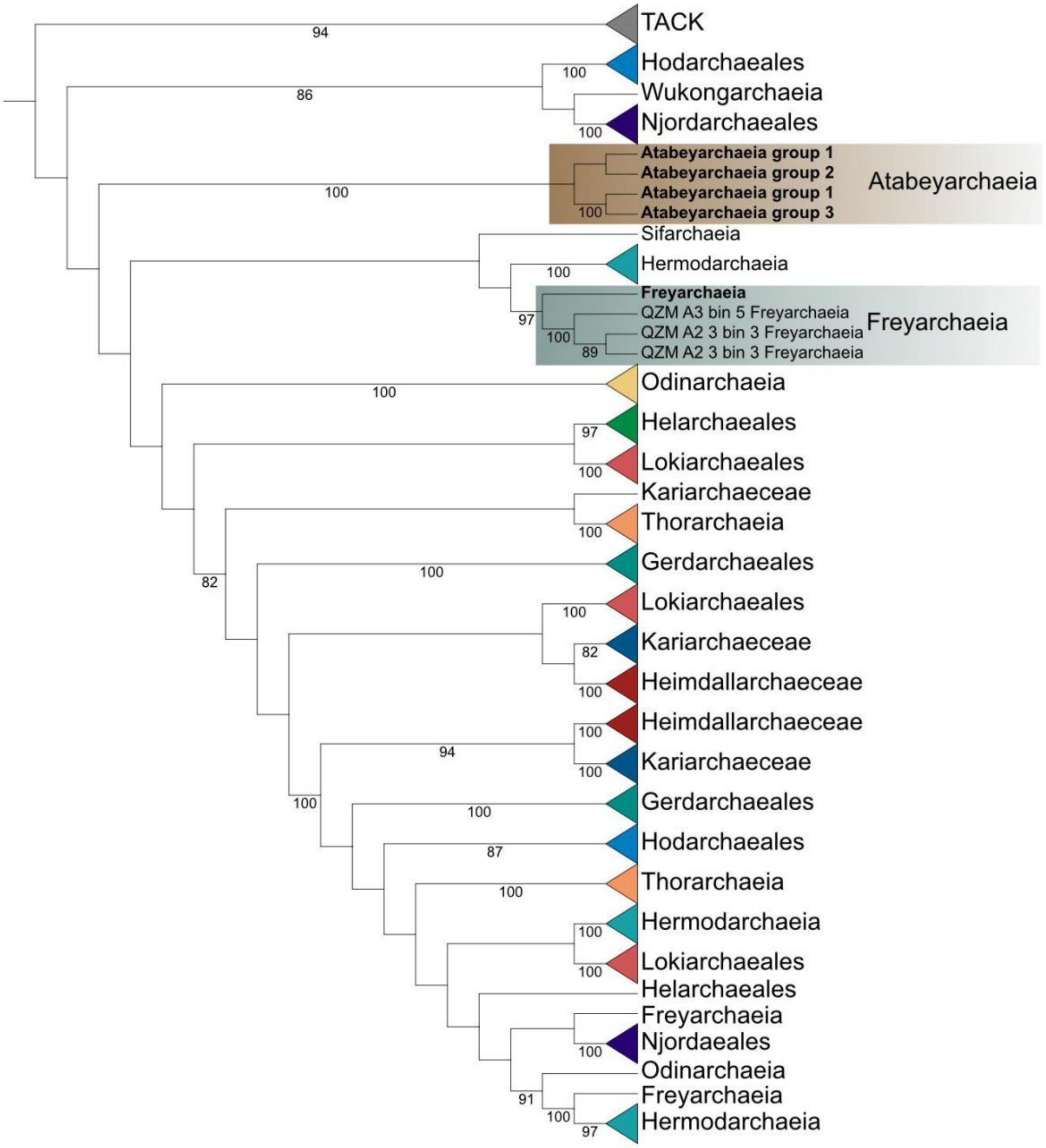


**Supplementary Figure 4** Maximum-likelihood tree, inferred with IQtree and the best-fit GTR+F+R6 model, using 16S ribosomal proteins from Asgard and TACK. Ultrafast bootstrap support values of >80 are shown.

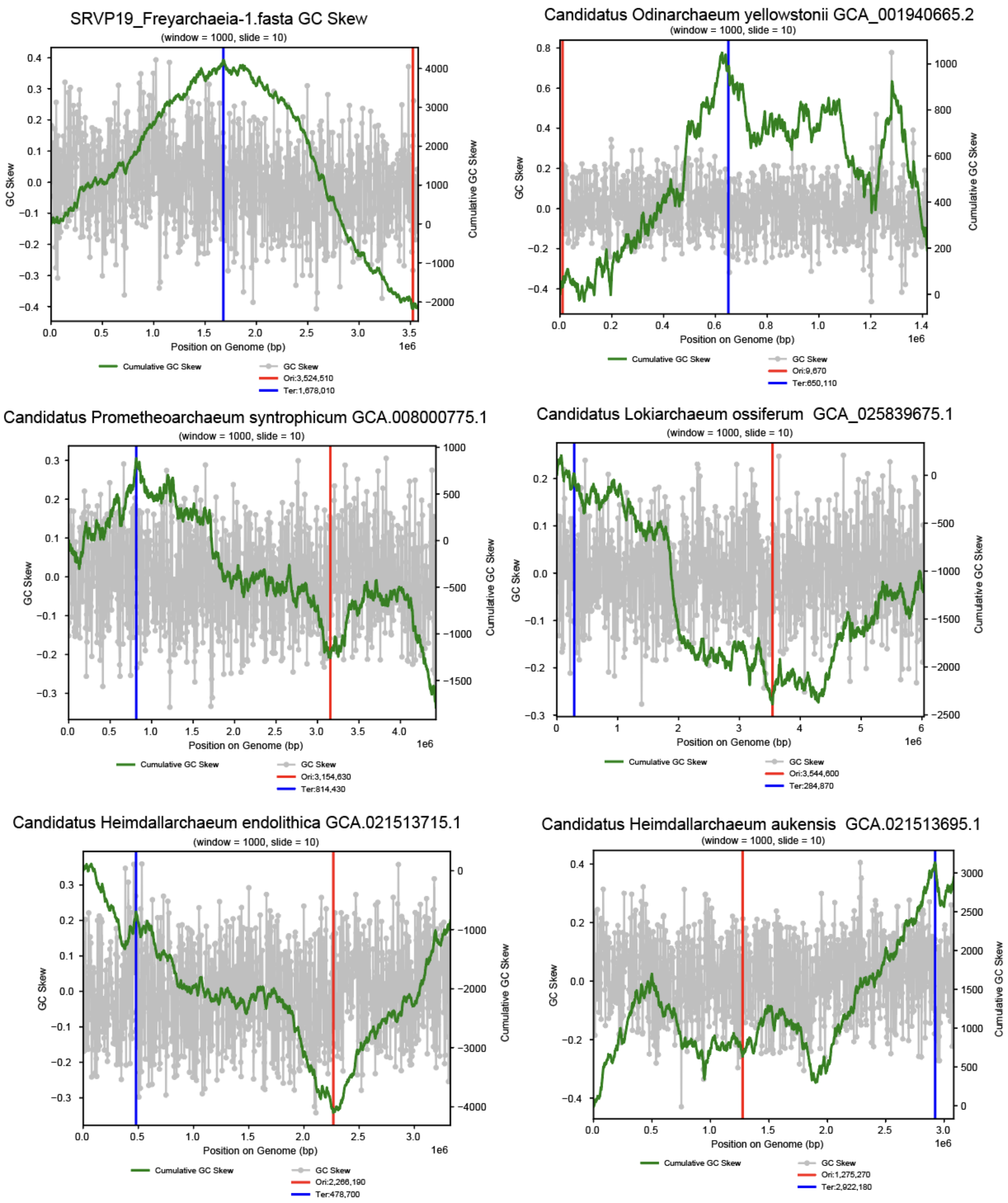


**Supplementary Figure 5** Cumulative GC skew of published Asgard complete genomes. The GC skew is shown as a gray plot and the cumulative GC skew is overlain (green line).

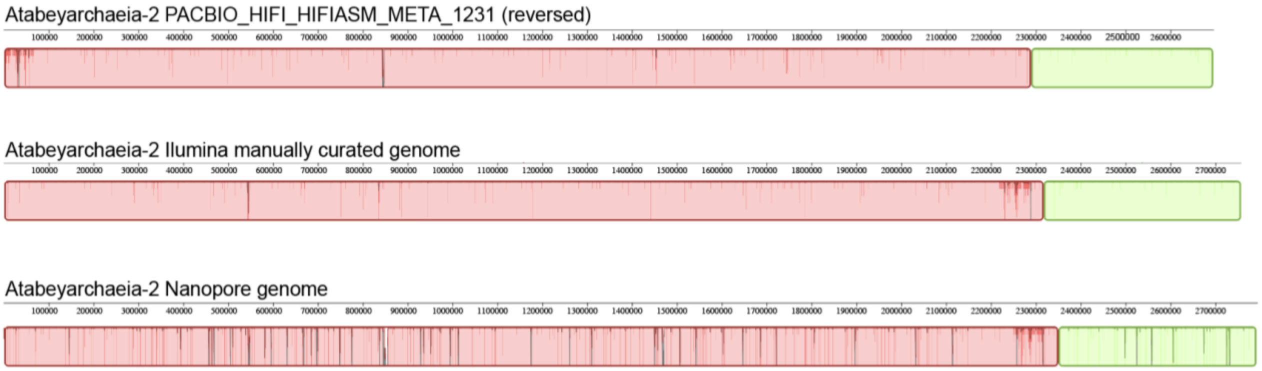


**Supplementary Figure 6** Confirmation of complete Atabeyarchaeia genome architecture reported based on Illumina assemblies. Overall topology of genomes for Atabeya-2 PacBio assembled circular genome, Illumina manually curated genome and assembled nanopore genome. The green sequences have been reversed to correct orientations, completing the genome.

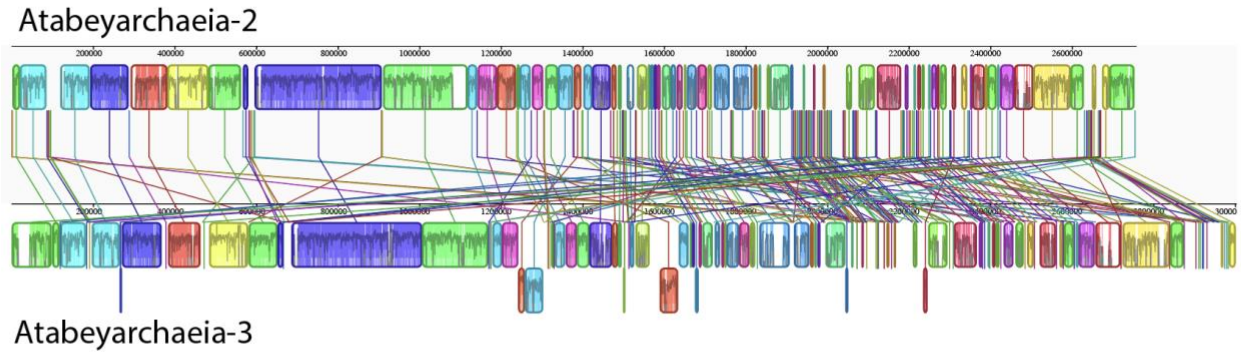


**Supplementary Figure 7** Nanopore assembled circular contig revealed another Atabeyarchaeia group-3.

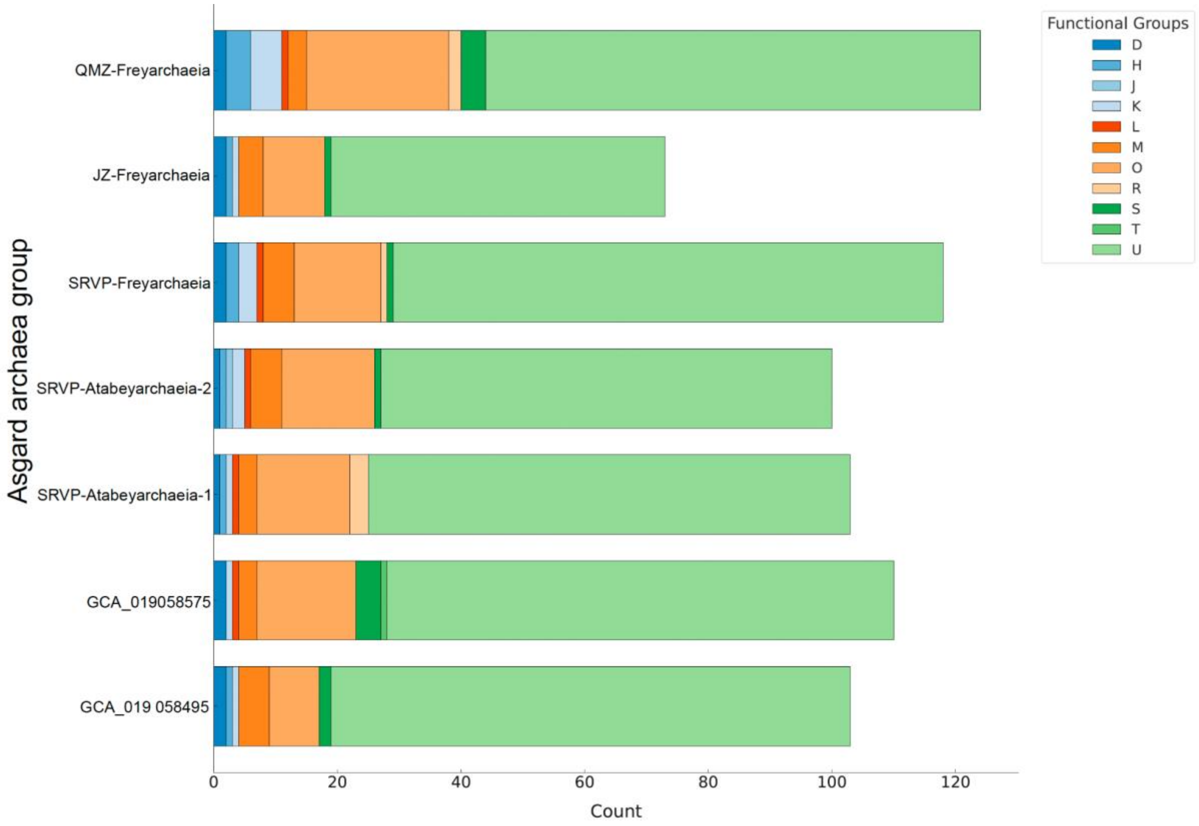


**Supplementary Figure 8** Distribution of eukaryotic signature proteins (ESPs) present in Atabeyarchaeia and Freyarchaeia genomes used in this study. ArcCOGs functional categories are J, Translation, ribosomal structure, and biogenesis; L, Replication, recombination, and repair. K, Transcription; L, Replication, recombination, and repair. M, Cell wall/membrane/envelope biogenesis; R, General function prediction only; S, Function unknown. O, Post-translational modification, protein turnover, and chaperones; H, Coenzyme transport and metabolism.

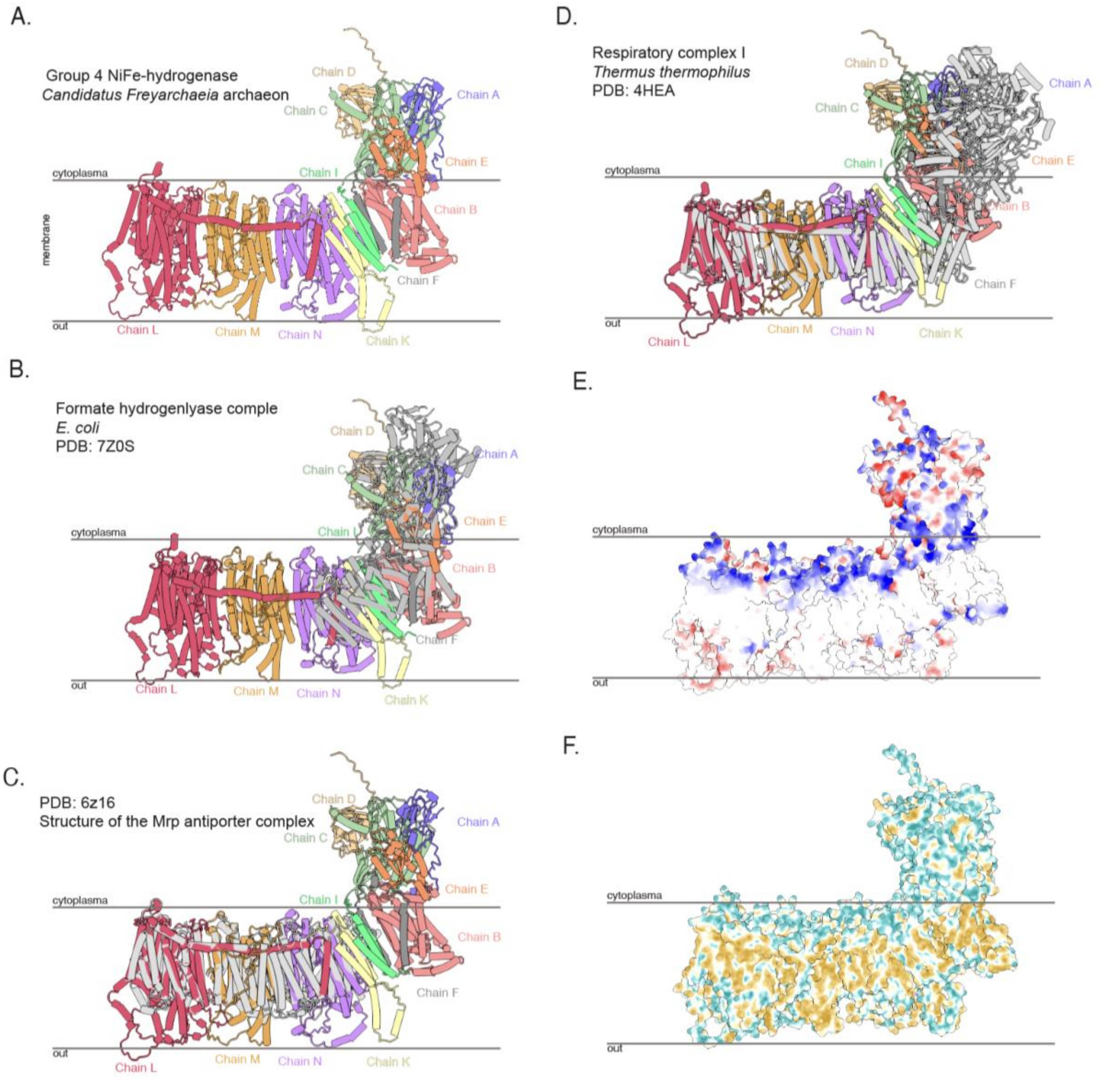


**Supplementary Figure 9** Structural superimposition of characterized complexes. (a) AlphaFold multimer models of [NiFe]-hydrogenase module and the proton-translocating membrane module where each candidate subunit is represented by a different color based on the best subunit matched from Atabeyarchaeia-1. (b) Superimposition of the formate hydrogenlyase complex from E. coli. (c) Superimposition of the Mrp antiporter complex. (d) Superimposition of the respiratory complex I hydrogenase from *Thermus thermophilus*, with hydrophobic regions marked in orange to denote areas rich in hydrophobic amino acids. (e) Model of the complex with surface electrostatic potential coloration: blue for positively charged regions, red for negatively charged areas, and white for neutral zones. (f) This color scheme elucidates the charge distribution on the protein's surface, providing insights into potential interactions with other biomolecules or ions

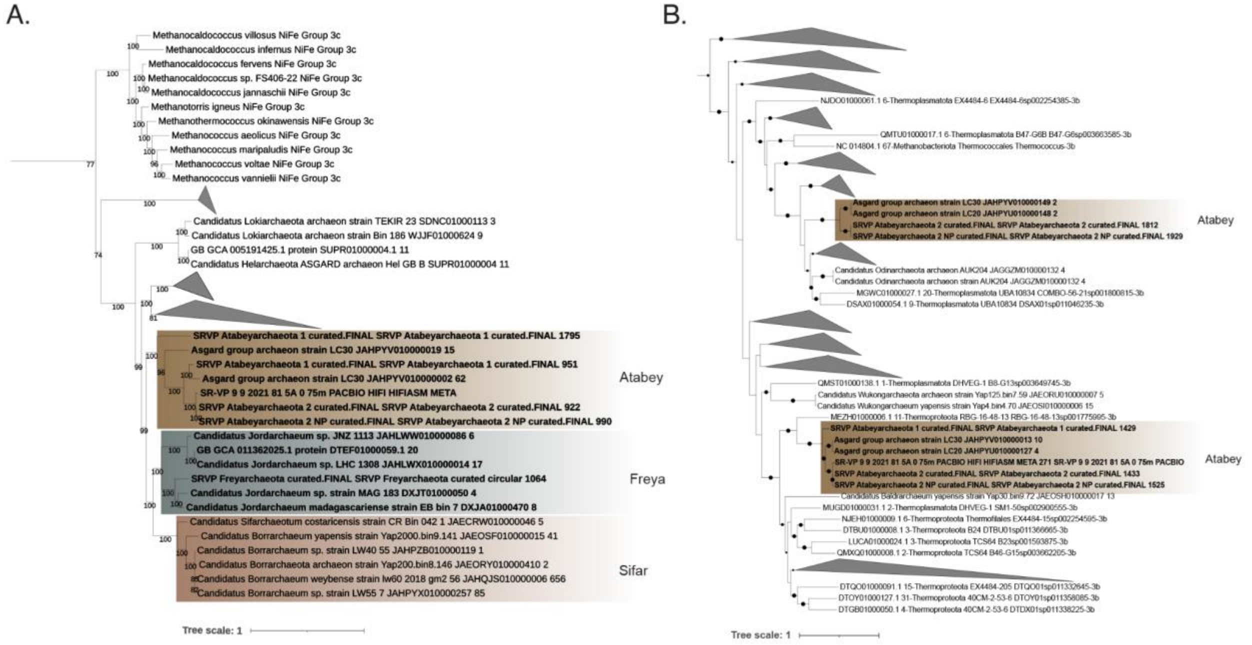


**Supplementary Figure 10** Maximum-likelihood phylogenetic reconstructions of the large subunit of group 3 [NiFe]-hydrogenases. (a) Group 3c (b) Group 3b The trees were generated with IQ-TREE v2.0.7, model LG+F+R10 was chosen according to BIC. Ultrafast bootstrap support values of >90 are shown.

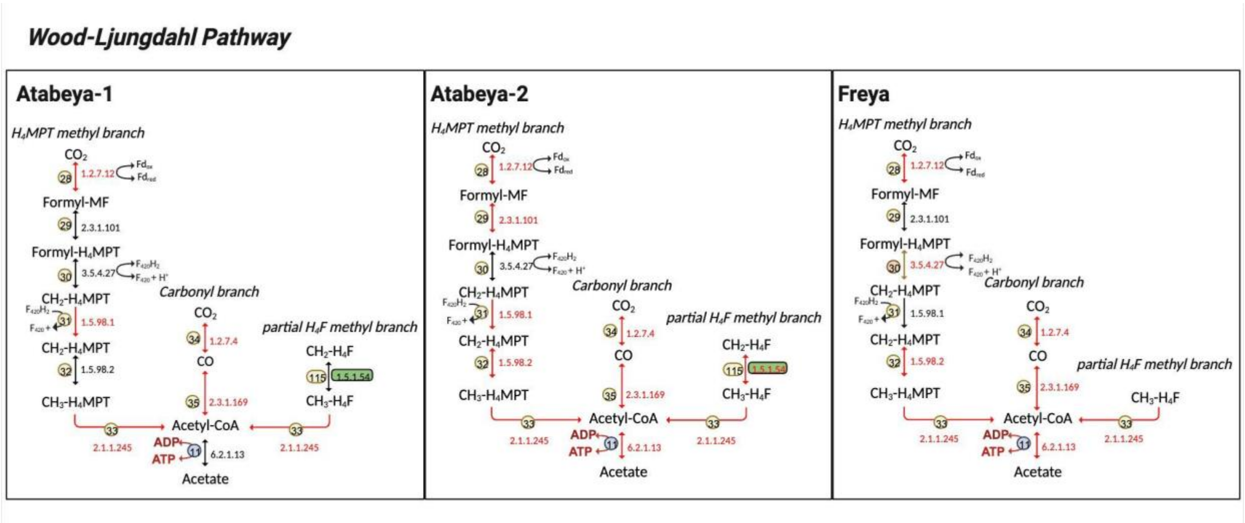


**Supplementary Figure 11** Genes and transcripts for the Wood-Ljungdahl Pathway (WLP). The gene numbers in circles correspond to those in Fig. 2 and supplementary table 8. Red arrows indicate that transcripts were detected. Step 115 is unique to Atabeyarchaeia. Created using BioRender.com.

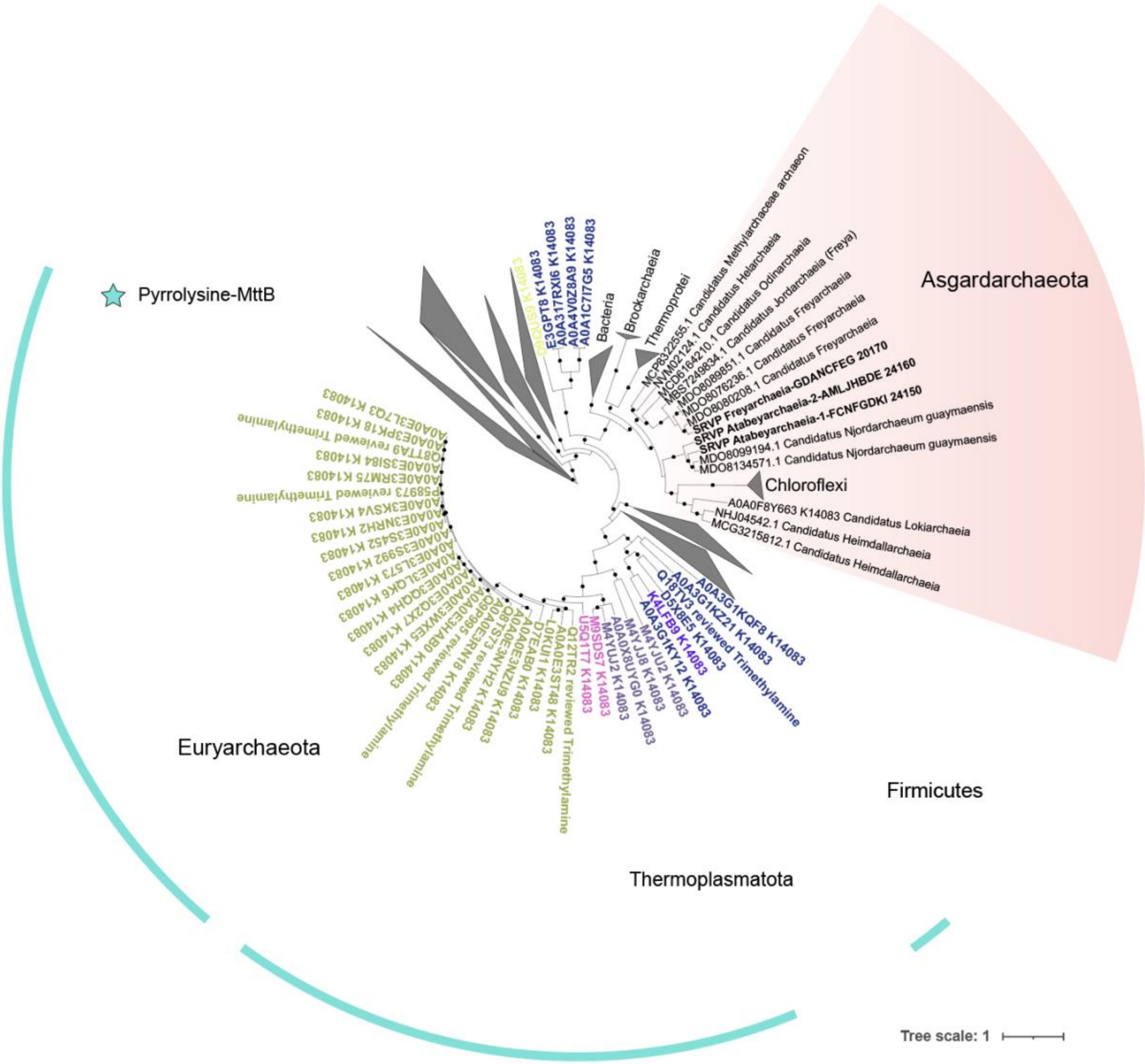


**Supplementary Figure 12** Non-Pyl trimethylamine methyltransferase homologs are widespread in Asgardarchaeota Maximum likelihood phylogeny of non-Pyl trimethylamine methyltransferase A subunit homologs (MttB). We identified two Atabeyarchaeia and two Freyarchaeia MttB-like in the complete genomes, which were aligned with 800 references[117] and 25 additional blastp hits. The outer light blue ring highlights pyrrolysine-containig reference enzymes. The colored leaf names display the taxonomic information from the UnitProt archaeal (olive- Euryarchaeota; pink- Methanomassiliicoccales; light purple- unclassified Thermoplasmatales) and Clostridial references (blue- Eubacteriales; bright purple- Thermoanaerobacterales; yellow- Halanerobiales). The red color range highlights the range of sequences from Asgardarchaeota, which span either side of the Chloroflexi group. The sequences were aligned and trimmed with MAFFT v7.505 and trimAl v1.4.rev15. The tree was generated with IQ-TREE v.1.6.12, model LG+C20+R+F. Ultrafast bootstrap support values of >80 are shown.

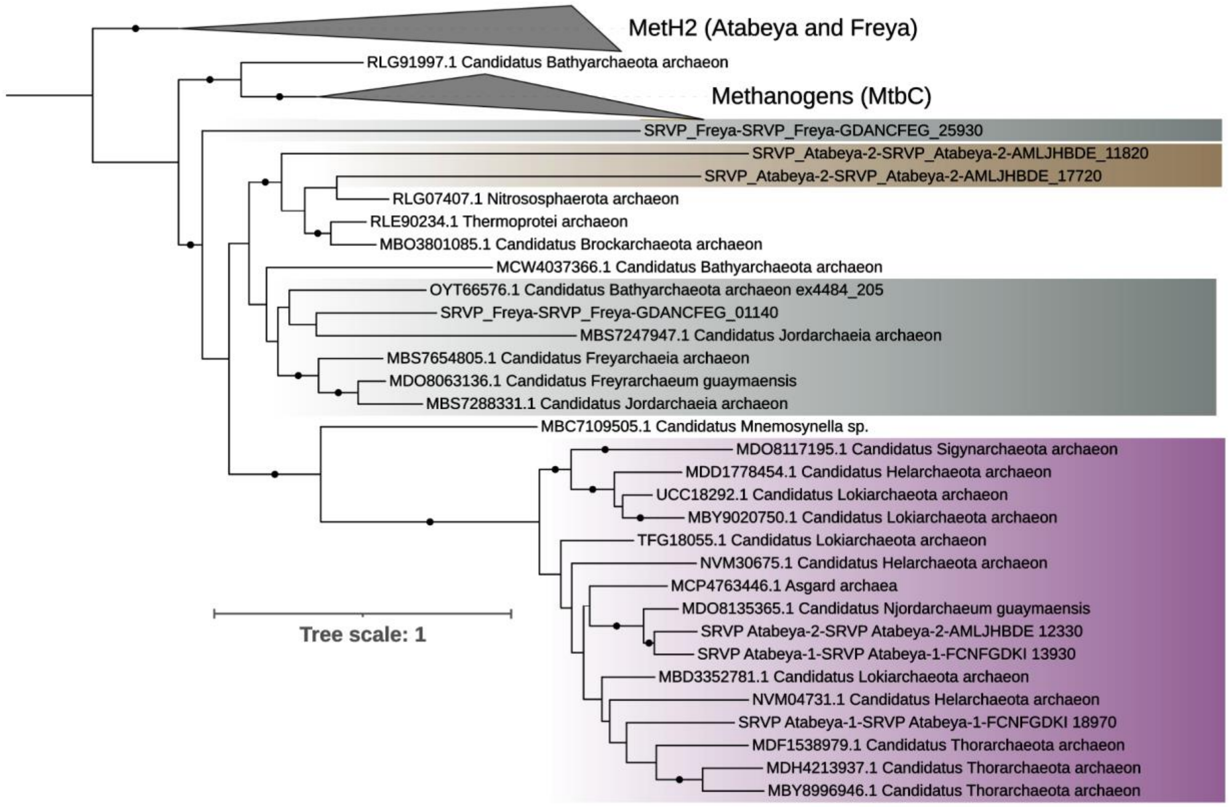


**Supplementary Figure 13** Non-Py MtbC dimethylamine-specific corrinoid protein (MtbC) homologs Maximum likelihood phylogeny of Non-Py dimethylamine-specific corrinoid protein homologs (MtbC and MtbC-like). We identified two Atabeyarchaeia and two Freyarchaeia MtbC-like in the complete genomes, which were aligned with a subset of 10 references based on protein identity from Methanogenic archaea and 25 additional blastp hits. The sequences were aligned and trimmed with MAFFT v7.505 and trimAl v1.4.rev15. The tree was generated with IQ-TREE v.1.6.12, model LG+C20+R+F. Ultrafast bootstrap support values of >80 are shown.

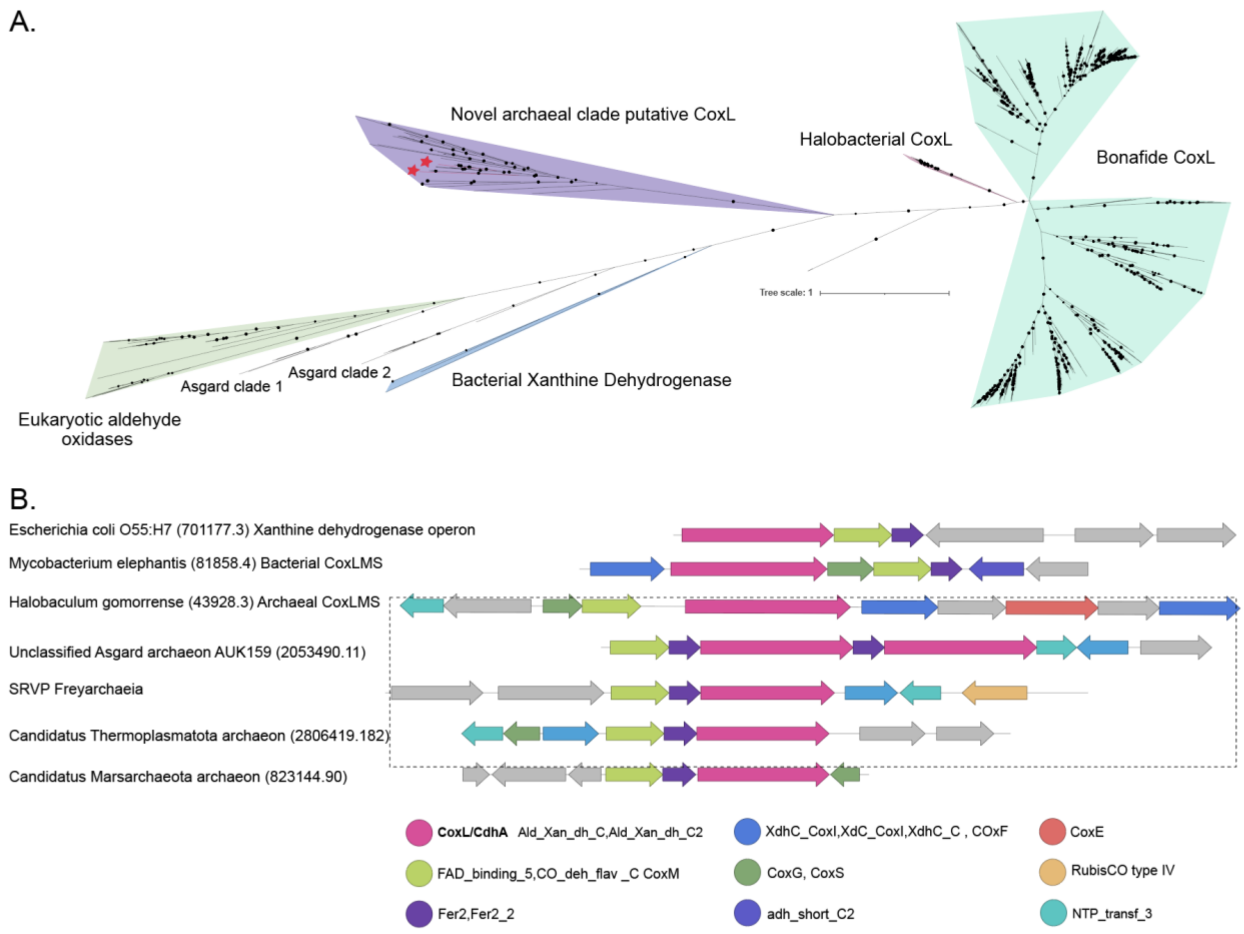


**Supplementary Figure 14** Novel archaeal aerobic carbon monoxide dehydrogenase in Asgard archaea. (a) Maximum likelihood phylogenetic tree of the aerobic carbon monoxide dehydrogenase large subunit (CoxL) sequences identified in Asgard archaea. It includes a comparison with reference sequences from established categories such as bonafide CoxL type I and II, halobacterial CoxL, as well as bacterial xanthine dehydrogenase (XDH), and aldehyde oxidases from eukaryotes as well as Asgard archaea. This phylogeny elucidates the evolutionary relationship and potential functional diversification of CoxI within these groups. (b) The panel presents the genetic architecture of cox genes genes with gene clusters color-coded based on Pfam domain classifications and gene annotations. This organization highlights the structural variability and potential regulatory elements within the genomic context of these archaeal enzymes. Ultrafast bootstrap support values of >80 are shown.

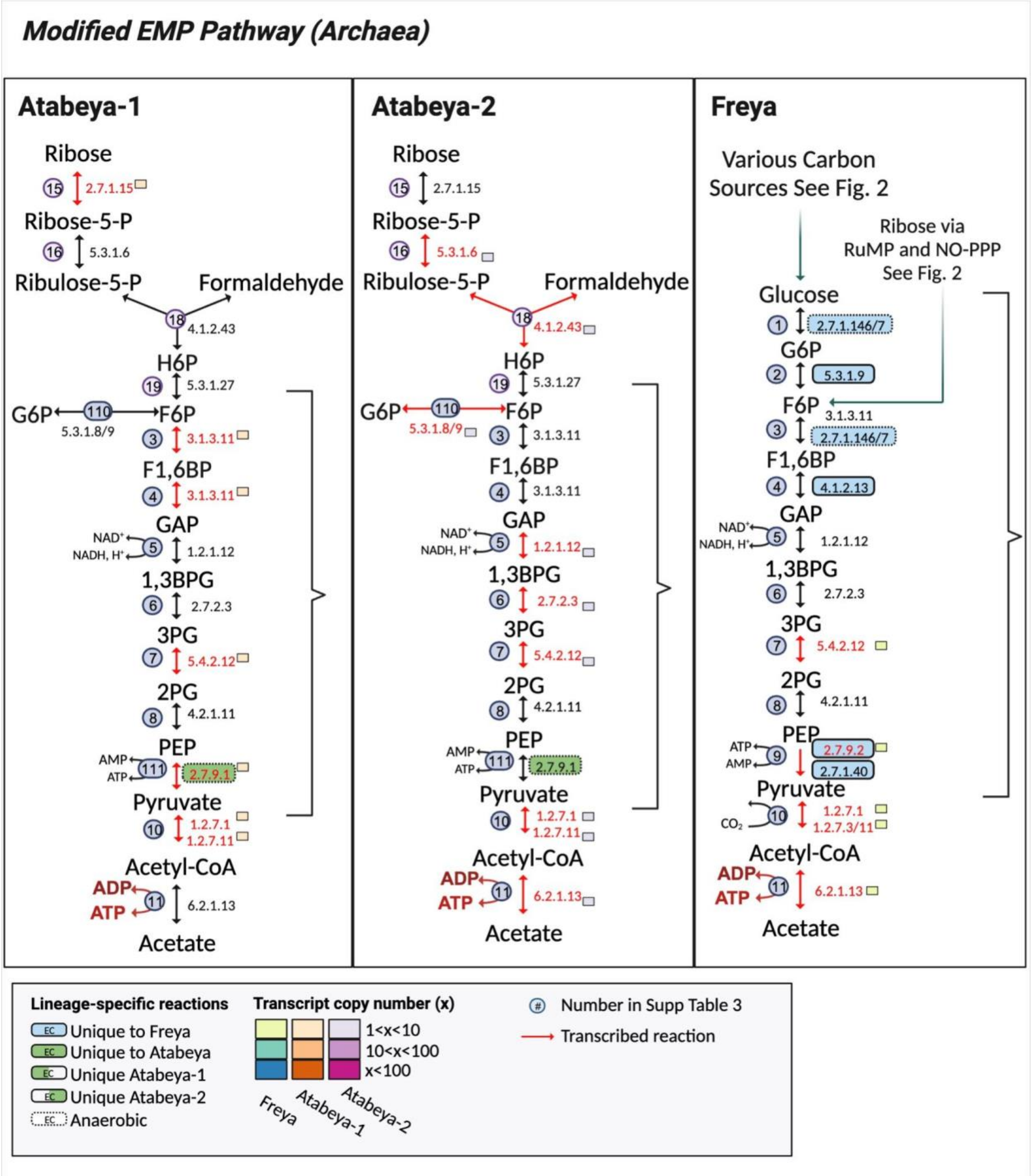


**Supplementary Figure 15** Transcriptome map of the archaeal modified Embden-Meyerhof-Parnas (EMP) Pathway for Atabeyarchaeia (Atabeya-1 and Atabeya-2) and Freyarchaeia (Freya). The numbers correspond to those in Fig. 2 and Supplementary Table 7. Red arrows indicate mapped transcripts, the rectangles show the number of transcripts, dashes around EC numbers show oxygen-sensitive enzymes, and the green boxes behind the EC numbers indicate lineage-specific reactions for Atabeyarchaeia genomes, matching Fig. 2. Created using BioRender.com.

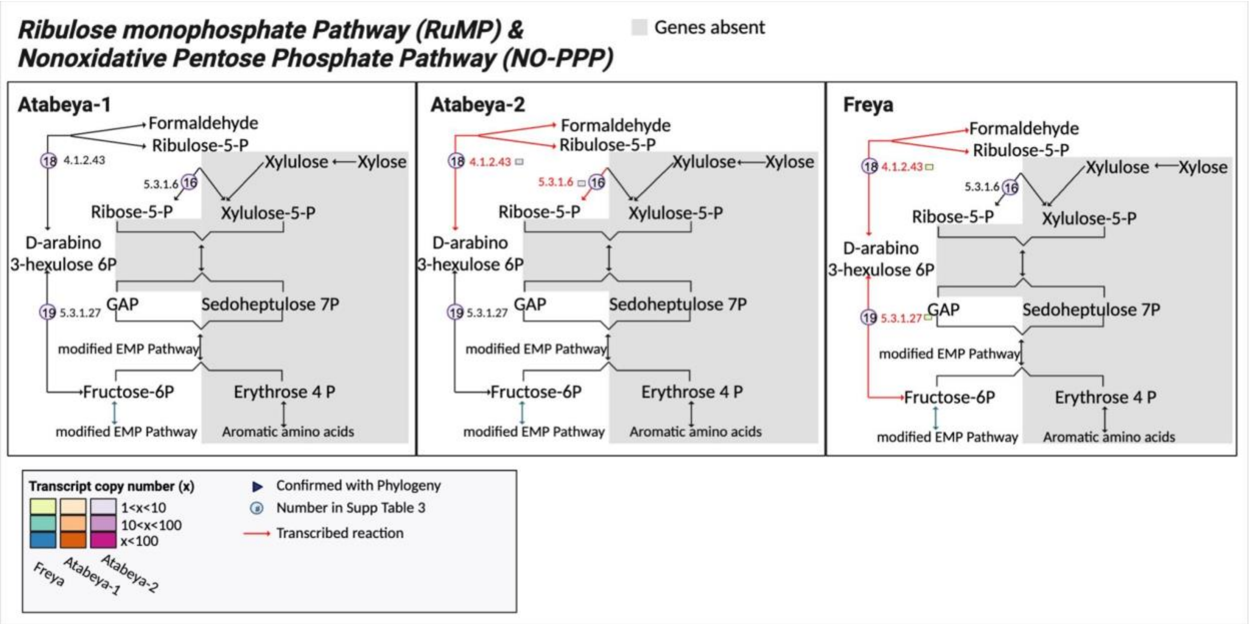


**Supplementary Figure 16** Transcriptome map of the Ribulose Monophosphate Pathway (RuMP) and the Nonoxidative Pentose Phosphate Pathway (NO-PPP) for Atabeyarchaeia (Atabeya-1 and Atabeya-2) and Freyarchaeia (Freya). The numbers correspond to those in Fig. 2 and Supplementary Table 7. Red arrows indicate mapped transcripts and the rectangles show the number of transcripts, matching Fig. 2. Created using BioRender.com.

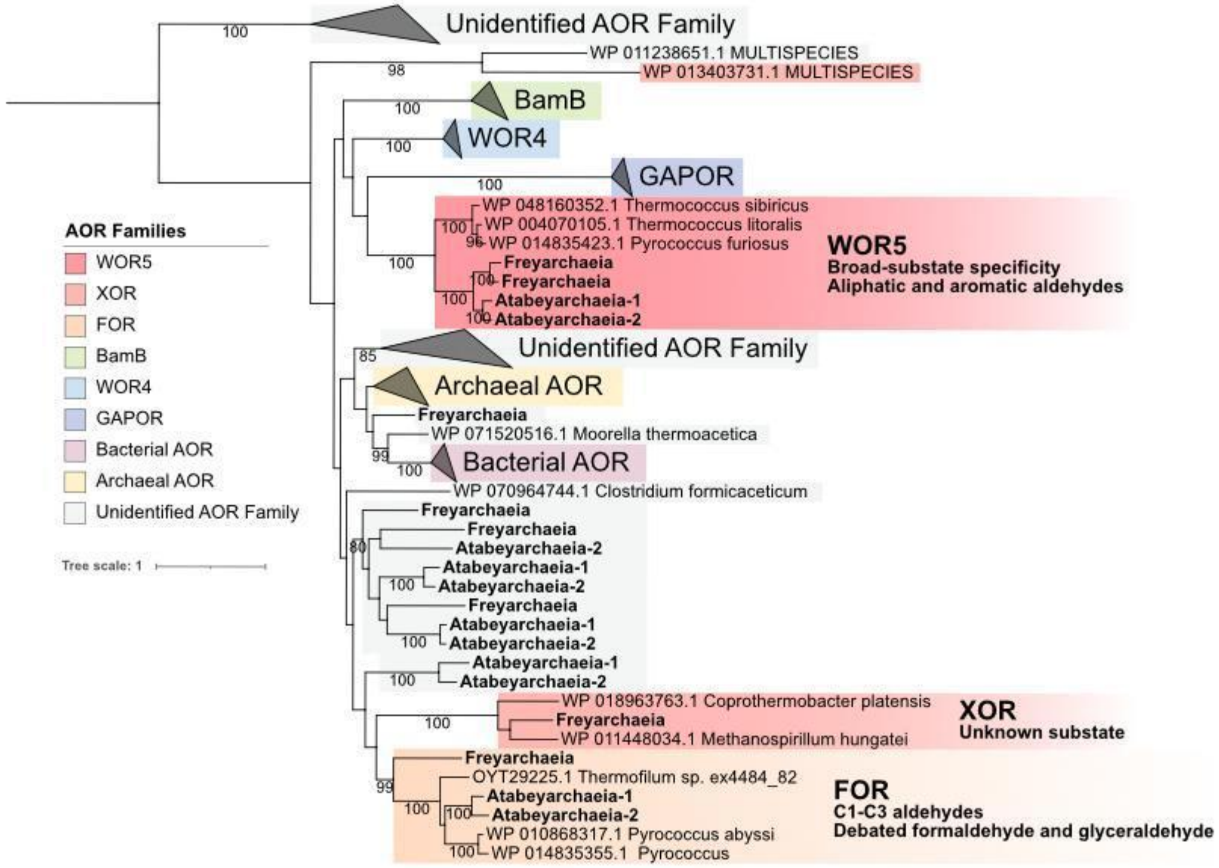


**Supplementary Figure 17** Maximum likelihood phylogeny of aldehyde oxidoreductases (AOR) to determine the specific family and potential substrate-specificity of the highlighted Atabeya-1, Atabeya-2, Freya sequences. The sequences for the three complete genomes were extracted and aligned with identified sequences from Arndt 2019. Mafft auto (v7.505) and trimAl -gt 0.5 (v1.4.rev15) were used for aligning and trimming, respectively. We used Iqtree (v1.6.12) to determine the maximum likelihood phylogeny, and LG+F+R4 was the model chosen according to BIC. Based on our analysis Freya and Atabeya sequences cluster with three known families (WOR5, XOR, and FOR) and two undescribed groups. Both Atabeyarchaeia genomes cluster with FOR and WOR5; whereas, Freya has sequences clustering with those groups and XOR. The family classification of Atabeyarchaeia and Freyarchaiea AORs is supported by the metabolism shown in **Fig. 2**.

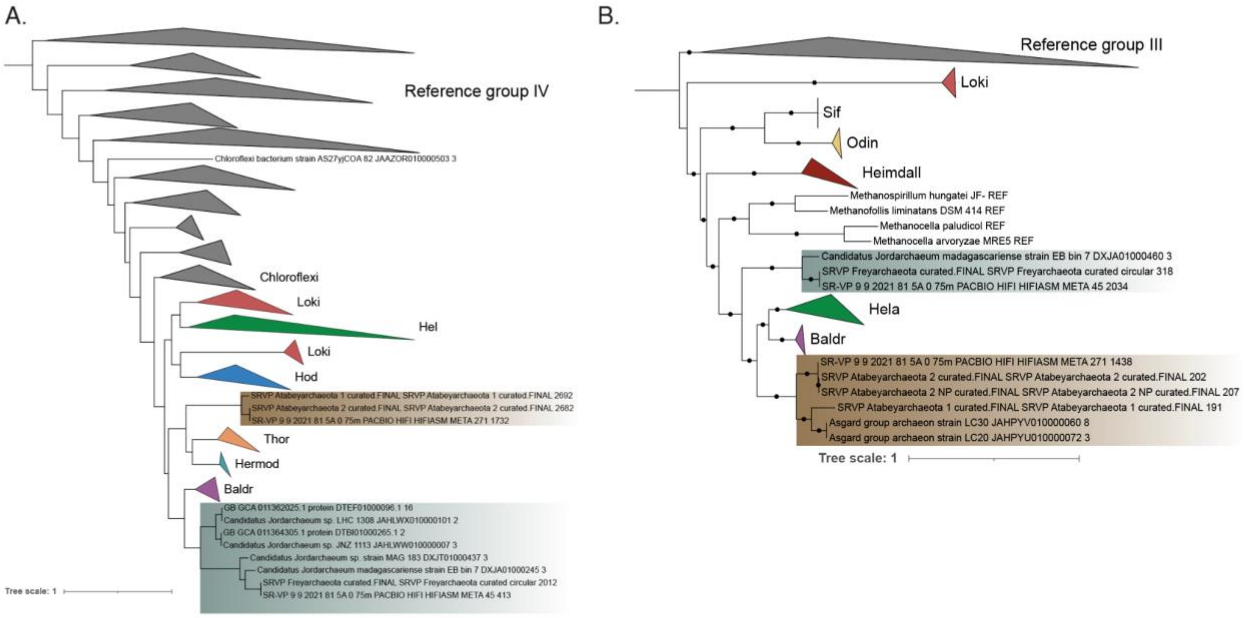


**Supplementary Figure 18** Maximum likelihood phylogeny of RubisCo-like proteins (group IV and methanogenic group III). We identified two Atabeyarchaeia and two Freyarchaeia RbcL-like in the complete genomes, which were aligned with a subset of 1,000 references.The sequences were aligned and trimmed with MAFFT v7.505 and trimAl v1.4.rev15. The tree was generated with IQ-TREE v.1.6.12, model LG+C20+R+F. Ultrafast bootstrap support values of >80 are shown.

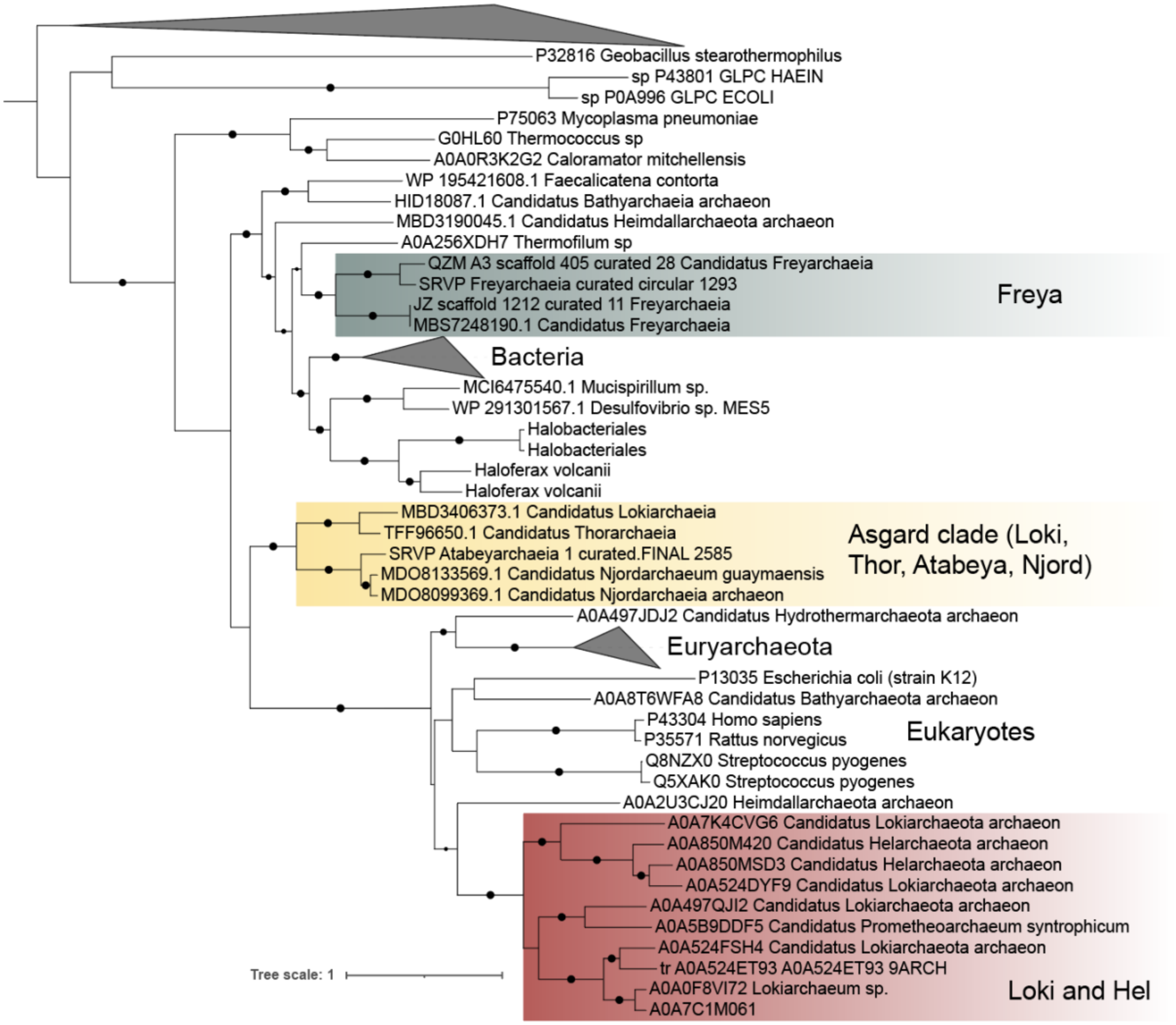


**Supplementary Figure 19** Maximum likelihood phylogeny of GlpA. We identified two Atabeyarchaeia and two Freyarchaeia GlpA proteins in the complete genomes, which were aligned with a subset of 123 GlpA references from uniprot and 50 top hits from NCBI against the non redundant database in November 2023. The sequences were aligned and trimmed with MAFFT v7.505 and trimAl v1.4.rev15. The tree was generated with IQ-TREE v.1.6.12, model LG+C20+R+F. Ultrafast bootstrap support values of >80 are shown.

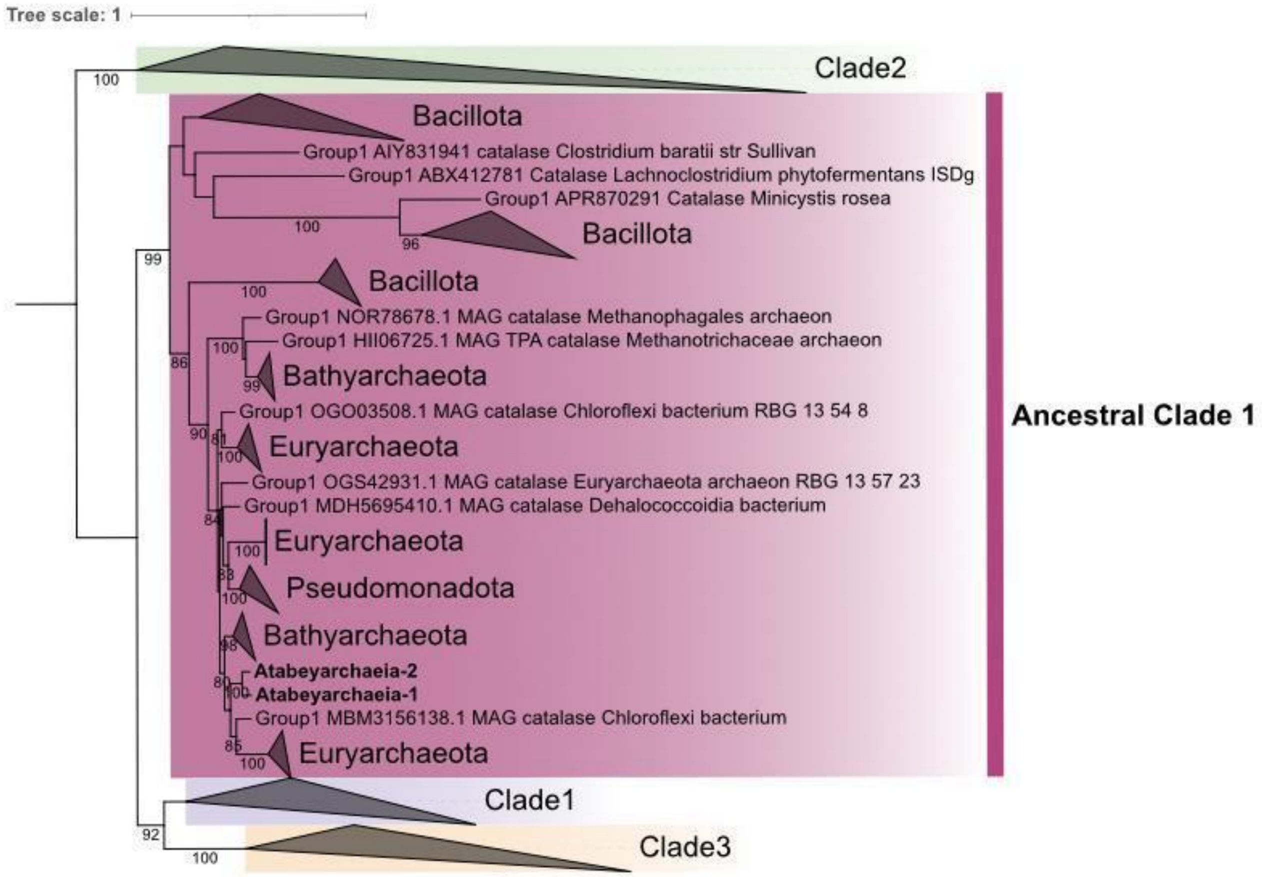


**Supplementary Figure 20** Maximum likelihood phylogeny of catalases to determine the specific clade of the Atabeyarchaeia-1 and Atabeyarchaeia-2 sequences. The Freyarchaeia catalase was removed after several iterations from the tree as it was a partial sequence, grouping with Atabeyarchaeia-1 and Atabeyarchaeia-2 sequences. Initially, catalase clades were determined by sub-setting the bacterial typical catalase sequences in https://doi.org/10.3389/fmicb.2021.645477 by group with MMSeq2 and aligning the subset with Mafft auto (v7.505). I augmented this alignment with reviewed sequences from IPR024711 (clade 1&3) and IPR024712 (clade 2). This expanded set of sequences was aligned with Mafft auto (v7.505) and trimmed with trimAl -gt 0.5 (v1.4.rev15). After several rounds of manually checking the alignment, we used Iqtree (v1.6.12) to produce the maximum likelihood phylogeny, and LG+R8 was the model chosen according to BIC. The clade classification of Atabeyarchaeia and Freyarchaeia catalase hits supported the annotations in **Supplementary Data 7**. Ultrafast bootstrap support values of >80 are shown.

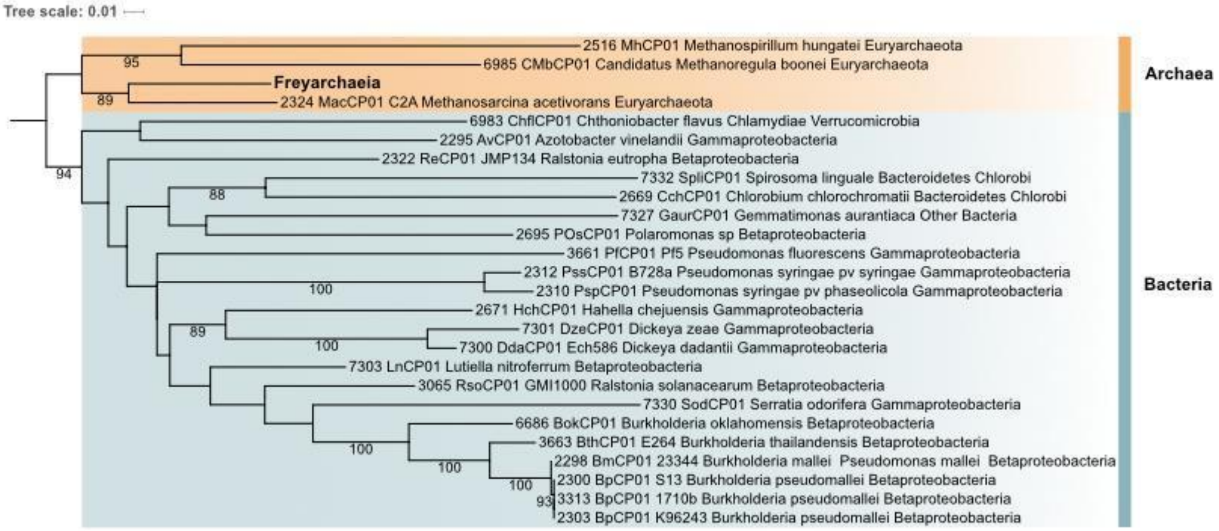


**Supplementary Figure 21** Maximum likelihood phylogeny to check annotation of catalase peroxidase in Freyarchaeia. The highlighted Freya sequence was blasted against those peroxidase sequences in the PeroxiBase Database in October 2023. These sequences were downloaded, aligned with Mafft auto (v7.505), and trimmed with trimAl -gt 0.5 (v1.4.rev15). Iqtree (v1.6.12) was used to produce the maximum likelihood phylogeny, and WAG+I+G4 was the model chosen according to BIC. The clade classification of the Freyarchaeia catalase peroxidase hit was supported by the annotations in Table S7. Ultrafast bootstrap support values of >80 are shown.

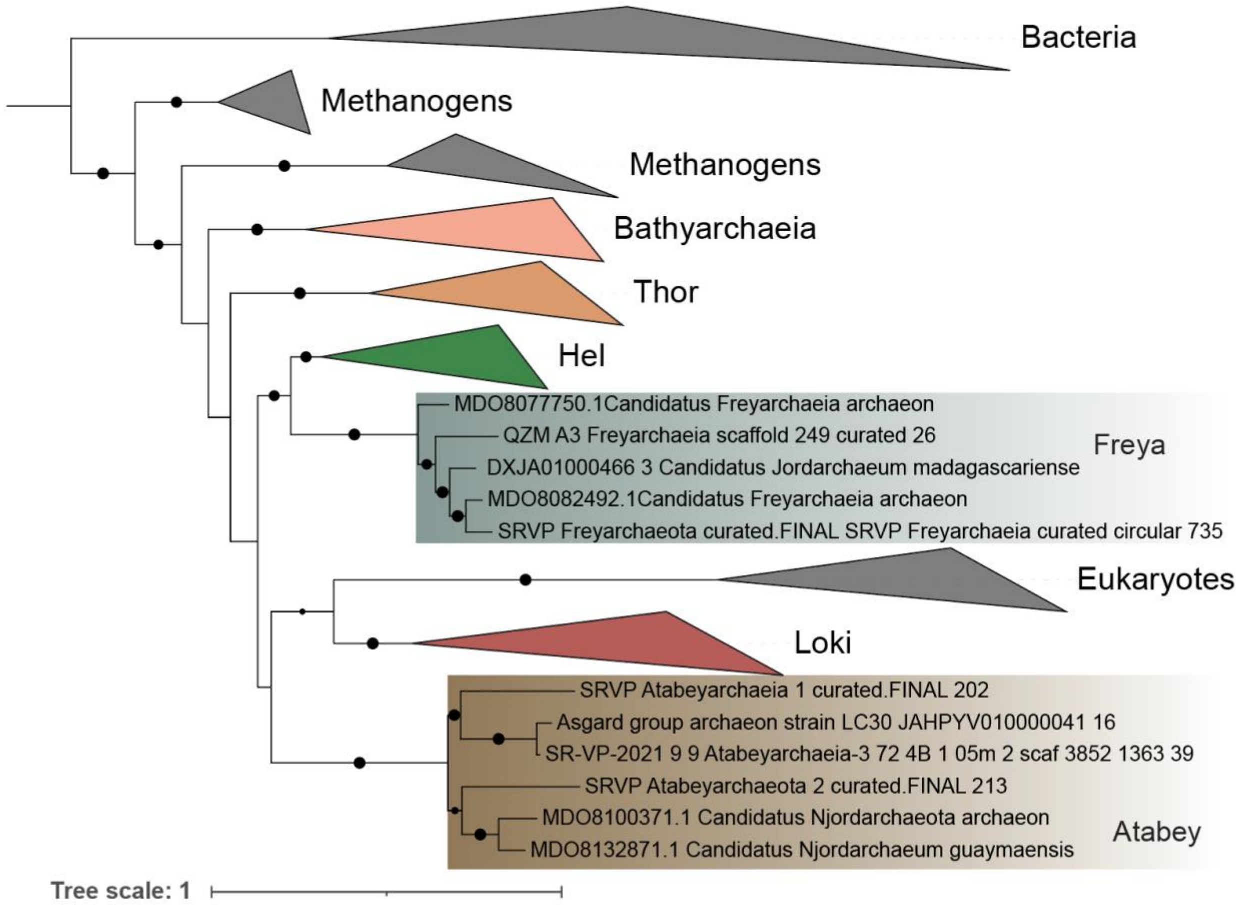


**Supplementary Figure 22** Phylogenetic analysis shows that the Sec elongation factor sequences from Atabeyarchaeia and Freyarchaeia are closely related to other Asgard members and Eukaryotes. Ultrafast bootstrap support values of >90 are shown.

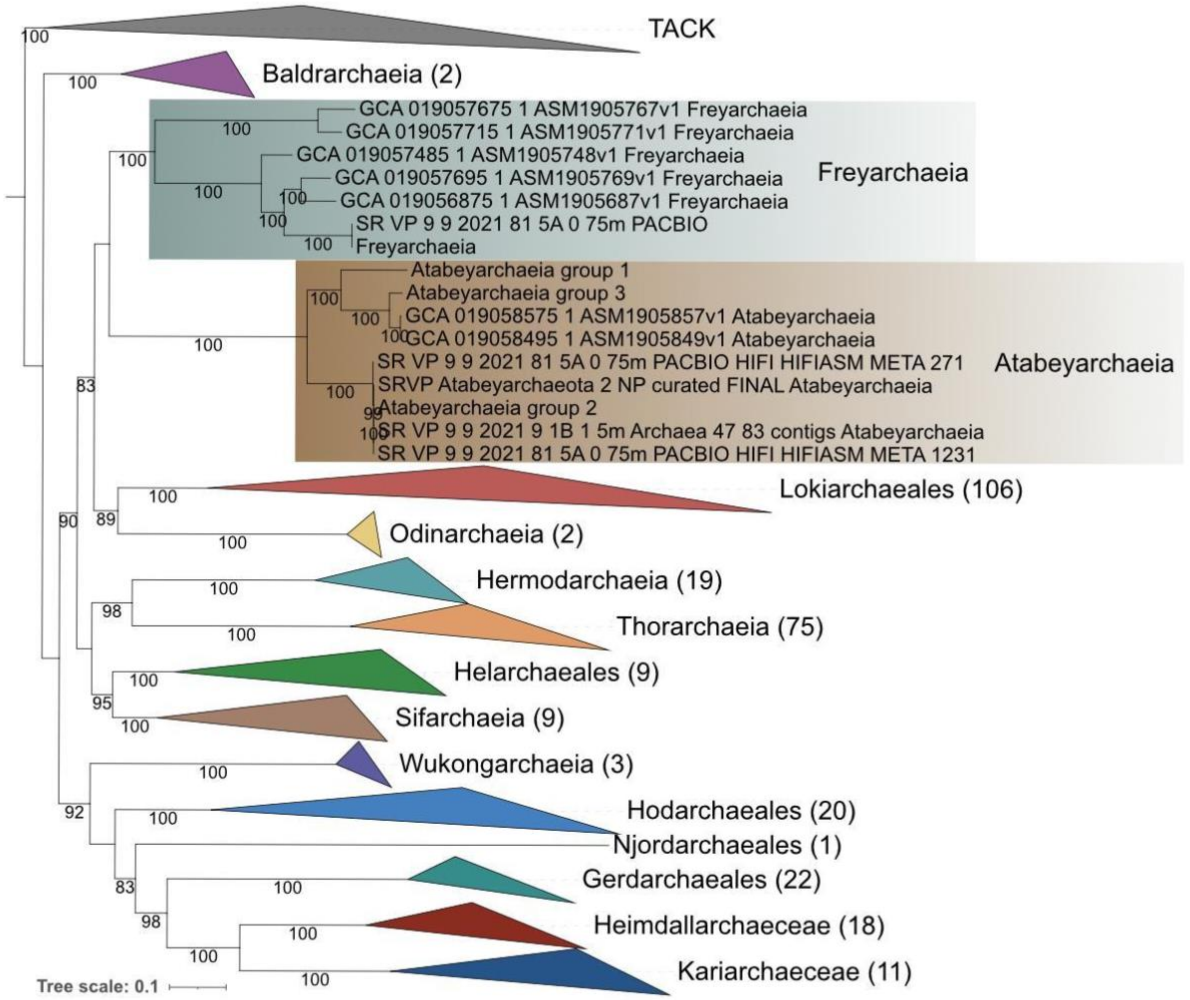


**Supplementary Figure 23** Maximum-likelihood tree, inferred with IQtree and the best-fit LG+F+R10 model, using Phylosift 37 markers from Asgardarchaeota and TACK. Ultrafast bootstrap support values of >80 are shown.

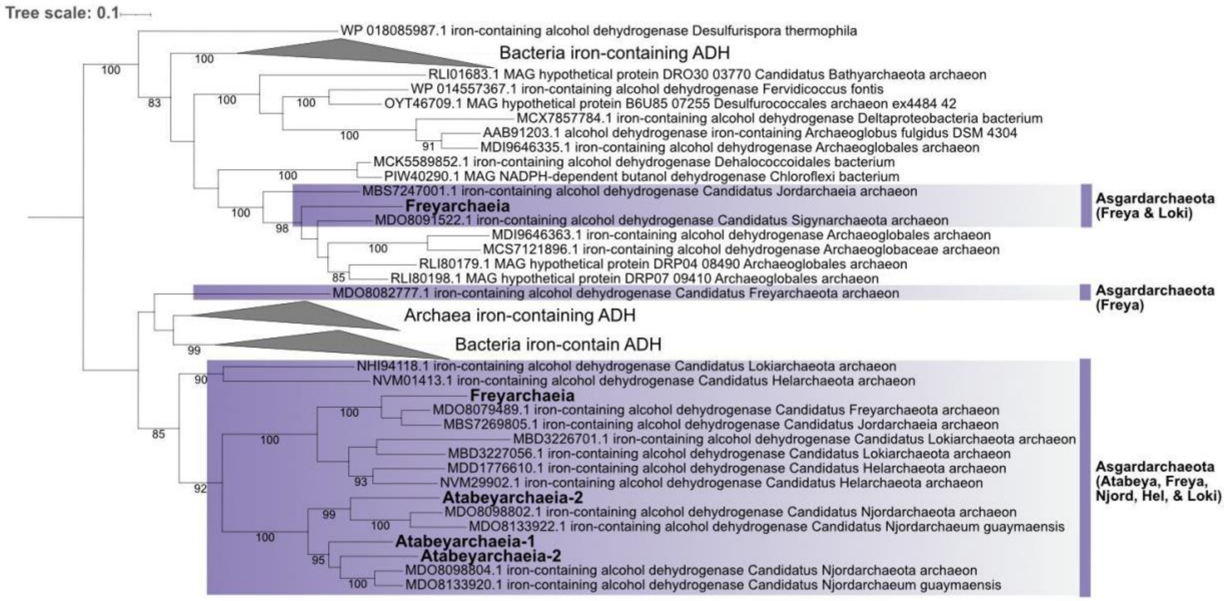


**Supplementary Figure 24** Maximum likelihood phylogeny of iron-containing alcohol dehydrogenases (ADH) in Atabeyarchaeia and Freyarchaeia complete genomes to determine potential substrate specificity of these enzymes. Freyarchaeia has a putative NADPH-dependent butanol dehydrogenase (BDH) similar to that in Chloroflexi bacterium (PIW40290), which is shown in **Fig. 2** and discussed in detail in the "**Supplementary text**" section. Ultrafast bootstrap support values of >80 are shown.

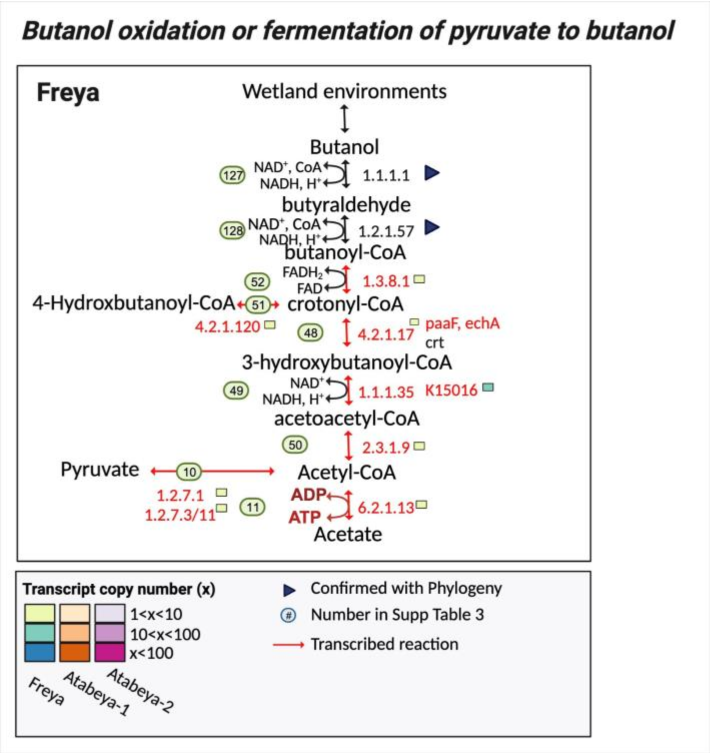


**Supplementary Figure 25** Transcriptome map of the proposed butanol oxidation pathway for Freyarchaeia. The numbers correspond to those in **Fig. 2** and Supplementary Table 7. Red arrows indicate mapped transcripts and the rectangles show the number of transcripts, matching **Fig. 2.** Created using BioRender.com.

107. Chastain, C. J. *et al.* Functional evolution of C(4) pyruvate, orthophosphate dikinase. *J. Exp. Bot.* **62**, 3083–3091 (2011).
108. Buckel, W. & Barker, H. A. Two pathways of glutamate fermentation by anaerobic bacteria. *J. Bacteriol.* **117**, 1248–1260 (1974).
109. Williams, T. J., Allen, M., Tschitschko, B. & Cavicchioli, R. Glycerol metabolism of haloarchaea. *Environ. Microbiol.* **19**, 864–877 (2017).
110. Okamura-Ikeda, K., Ohmura, Y., Fujiwara, K. & Motokawa, Y. Cloning and nucleotide sequence of the gcv operon encoding the Escherichia coli glycine-cleavage system. *Eur. J. Biochem.* **216**, 539–548 (1993).
111. Lie, T. J. *et al.* Essential anaplerotic role for the energy-converting hydrogenase Eha in hydrogenotrophic methanogenesis. *Proc. Natl. Acad. Sci. U. S. A.* **109**, 15473–15478 (2012).
112. Pedroni, P. *et al.* Characterization of the locus encoding the [Ni-Fe] sulfhydrogenase from the archaeon Pyrococcus furiosus: evidence for a relationship to bacterial sulfite reductases. *Microbiology* **141 ( Pt 2)**, 449–458 (1995).
113. Greening, C. *et al.* Genomic and metagenomic surveys of hydrogenase distribution indicate H2 is a widely utilised energy source for microbial growth and survival. *ISME J.* **10**, 761–777 (2016).
114. Wagner, T., Koch, J., Ermler, U. & Shima, S. Methanogenic heterodisulfide reductase (HdrABC-MvhAGD) uses two noncubane [4Fe-4S] clusters for reduction. *Science* **357**, 699–703 (2017).
115. Müller, V. New Horizons in Acetogenic Conversion of One-Carbon Substrates and Biological Hydrogen Storage. *Trends Biotechnol.* **37**, 1344–1354 (2019).
116. Cole, S. T. *et al.* Nucleotide sequence and gene-polypeptide relationships of the glpABC operon encoding the anaerobic sn-glycerol-3-phosphate dehydrogenase of Escherichia coli K-12. *J. Bacteriol.* **170**, 2448–2456 (1988).
117. Bojanova, D. P. *et al.* Well-hidden methanogenesis in deep, organic-rich sediments of Guaymas Basin. *ISME J.* **17**, 1828–1838 (2023).


**Formatted:** Line spacing: Multiple 1.15 li
