# Supplementary material for: Asgard archaea modulate potential methanogenesis substrates in wetland soil
Source: Nat Commun. 2024 Jul 31;15:6384. doi: 10.1038/s41467-024-49872-z (PMC11291895; doi:10.1038/s41467-024-49872-z)
Supplement: Supplementary file 3 — Description of Additional Supplementary Files [file 41467_2024_49872_MOESM3_ESM.pdf]

Description of Additional Supplementary Files for:

Asgard archaea modulate potential methanogenesis substrates in wetland soil

Supplementary Data Legends

File Name: Supplementary Data 1.
Description: Overall genomic statistics of complete genomes, MAGs and 245 public Asgard reference genomes downloaded from Bacterial and Viral Bioinformatics Resource Center (BV-BRC), database on March 20th, 2022 BCR.

File Name: Supplementary Data 2.
Description: Average amino acid identity (AAI) comparison of Atabeyarchaeia and Freyarchaeia genomes and phylogenetically related Asgardarchaeota phyla.

File Name: Supplementary Data 3.
Description: GTDB version 2.3.0: These results shows our genomes but also publicly available genomes used for the comparative genomic analyses

File Name: Supplementary Data 4.
Description: The tRNA genes and introns of Atabeyarchaeia and Freyarchaeia genomes.

File Name: Supplementary Data 5.
Description: Eukaryotic signature proteins in Atabeyarchaeia and Freyarchaeia

File Name: Supplementary Data 6.
Description: This table presents the RNA hits expressed as Reads Per Kilobase of transcript, per Million mapped reads (RPKM), for each genome analyzed. It is used to calculate expression levels across the complete genomes.

File Name: Supplementary Data 7.
Description: Hydrogenases present in the complete genomes

File Name: Supplementary Data 8.
Description: Metabolism sheet: Cell Diagram sheet includes key metabolic genes identified in Atabeyarchaeia and Freya complete genomes shown in Figure 2 metabolic overview. The sheet includes information about number of identified gene copies, metatranscript copy number, psortv 3.0.3 locations, and if there is a corresponding supplementary phylogeny. The next nine sheets contain raw annotations for KofamKOALAv1.3.0, Interproscanv5.6.1-93.0-64, MEBsv2_Pfamv34.0, HADEG_Alkanes, METABOLICv4, dbcan, MEROPSv12.4, DRAM, and HyDB

File Name: Supplementary Data 9.
Description: PSI-Blast results from ArCOGS

File Name: Supplementary Data 10.
Description: The identity and coordinates of the selenocysteine machinery in the Atabeyarchaeia and Freyarchaeia genomes.

File Name: Supplementary Data 11.
Description: The identity and coordinates of the selenoproteins and their insertion sequences in the Atabeyarchaeia and Freyarchaeia genomes.
