# Supplementary material for: Asgard archaea modulate potential methanogenesis substrates in wetland soil
Source: Nat Commun. 2024 Jul 31;15:6384. doi: 10.1038/s41467-024-49872-z (PMC11291895; doi:10.1038/s41467-024-49872-z)
Supplement: Supplementary file 5 — Reporting Summary [file 41467_2024_49872_MOESM5_ESM.pdf]

nature portfolio

Corresponding author(s): Jill F. Banfield

Last updated by author(s): Jun 5, 2024

Reporting Summary

Nature Portfolio wishes to improve the reproducibility of the work that we publish. This form provides structure for consistency and transparency in reporting. For further information on Nature Portfolio policies, see our Editorial Policies and the Editorial Policy Checklist.

Statistics

For all statistical analyses, confirm that the following items are present in the figure legend, table legend, main text, or Methods section.

n/a	Confirmed	
☐	☒	The exact sample size (*n*) for each experimental group/condition, given as a discrete number and unit of measurement
☐	☒	A statement on whether measurements were taken from distinct samples or whether the same sample was measured repeatedly
☐	☒	The statistical test(s) used AND whether they are one- or two-sided<br>*Only common tests should be described solely by name; describe more complex techniques in the Methods section.*
☒	☐	A description of all covariates tested
☒	☐	A description of any assumptions or corrections, such as tests of normality and adjustment for multiple comparisons
☐	☒	A full description of the statistical parameters including central tendency (e.g. means) or other basic estimates (e.g. regression coefficient) AND variation (e.g. standard deviation) or associated estimates of uncertainty (e.g. confidence intervals)
☒	☐	For null hypothesis testing, the test statistic (e.g. *F*, *t*, *r*) with confidence intervals, effect sizes, degrees of freedom and *P* value noted<br>*Give P values as exact values whenever suitable.*
☒	☐	For Bayesian analysis, information on the choice of priors and Markov chain Monte Carlo settings
☒	☐	For hierarchical and complex designs, identification of the appropriate level for tests and full reporting of outcomes
☒	☐	Estimates of effect sizes (e.g. Cohen's *d*, Pearson's *r*), indicating how they were calculated

Our web collection on statistics for biologists contains articles on many of the points above.

Software and code

Policy information about availability of computer code

Data collection

Geneious (v11.0.18+10) Licensed, paid version used in this study, free versions available)

IDBA_UD assembler
metaSPAdes
Bowtie2 v2.3.4.1
MetaBAT2
VAMB
MaxBin2 v1.1.3
ggKbase (https://ggkbase.berkeley.edu/)

Nanopore:
Guppy using the dna_r10.4.1_e8.2_sup@v3.5.1 model
porechop (v0.2.4)
fastp (v0.23.2)
with mappy (v2.24)
metaFlye (v2.9)
medaka consensus (v1.7.1)
Hapo-G (v1.3.1).

PacBio data:
Sequel II
BBDuk
hifiasm-meta

Data analysis	Databases used to analyze data and or against which data was compared:<br>Prodigal (v2.6.3)<br>KofamKOALA (v1.3.0)<br>HydDB (https://services.birc.au.dk/hyddb/)<br>PROKKA (v1.14.6)<br>MEBS (v2.0) (https://www.ebi.ac.uk/merops/)<br>Carbohydrate-Active enZYmes (CAZYmes) database (http://www.cazy.org/)<br>Metacyc Metabolic Pathway Database (https://metacyc.org/)<br>KEGG (https://www.genome.jp/kegg/)<br>COGs, arCOGs and asCOGs (ftp://ftp.ncbi.nih.gov/pub/wolf/COGs/arCOG)<br><br>Data was analyzed using the following published softwares:<br>DRAM<br>InterProScan (5.50-84.0)<br>METABOLIC (v4.0)<br>CheckM2<br>GTDB-Tk v2.3.0

For manuscripts utilizing custom algorithms or software that are central to the research but not yet described in published literature, software must be made available to editors and reviewers. We strongly encourage code deposition in a community repository (e.g. GitHub). See the Nature Portfolio guidelines for submitting code & software for further information.

Data

Policy information about availability of data

All manuscripts must include a data availability statement. This statement should provide the following information, where applicable:

- Accession codes, unique identifiers, or web links for publicly available datasets
- A description of any restrictions on data availability
- For clinical datasets or third party data, please ensure that the statement adheres to our policy

Prior to publication, the genomes reported in this study can be accessed via https://ggkbase.berkeley.edu/SRVP_asgard/organisms.

Research involving human participants, their data, or biological material

Policy information about studies with human participants or human data. See also policy information about sex, gender (identity/presentation), and sexual orientation and race, ethnicity and racism.

Reporting on sex and gender	N/A
Reporting on race, ethnicity, or other socially relevant groupings	N/A
Population characteristics	N/A
Recruitment	N/A
Ethics oversight	N/A

Note that full information on the approval of the study protocol must also be provided in the manuscript.

Field-specific reporting

Please select the one below that is the best fit for your research. If you are not sure, read the appropriate sections before making your selection.

[x] Life sciences   [ ] Behavioural & social sciences   [ ] Ecological, evolutionary & environmental sciences

For a reference copy of the document with all sections, see nature.com/documents/nr-reporting-summary-flat.pdf

Life sciences study design

All studies must disclose on these points even when the disclosure is negative.

Sample size	The choice of sample size was strategically made to ensure comprehensive coverage of the ecosystem, aiming for a wide-ranging collection of Asgard archaea genomes from wetland soils. Statistical methods were not used to set the sample size. Instead, we compiled data from multiple sites where Asgard genomes were likely to be present, to achieve thorough representation.
Data exclusions	No datasets or databases were excluded during our survey
Replication	We recovered genomes from Asgard archaea in 56 soil samples taken from the same site in Lake County, California, over the years 2017, 2019, 2020, and 2021. Each soil sample was processed separately to extract DNA, which was then individually assembled. We used Illumina, PacBio, and Oxford Nanopore sequencing to validate the complete genomes. This approach ensured that we accurately assembled the genomes from the DNA extracted. The DNA from each sample was sequenced and analyzed separately. Although we generally found unique genomes in each sample, some genomes appeared in more than one sample. We mapped sequencing reads from all samples to the most complete genomes to identify and quantify how often each genotype appeared across our samples.
Randomization	After collecting the samples from their natural enviroment, we mixed them thoroughly to minimize the impact of minor differences within the samples. We then extracted DNA from these mixed samples. Our study did not involve other randomization techniques because it focused on using DNA sequences to rebuild genomes, rather than conducting lab-based experiments.
Blinding	Blinding was not applicable in our research as it involved analyzing environmental samples. The nature of our study, focused on environmental sample collection and analysis, meant that the findings were not influenced by the outcomes of controlled trials, making blinding unnecessary.

Reporting for specific materials, systems and methods

We require information from authors about some types of materials, experimental systems and methods used in many studies. Here, indicate whether each material, system or method listed is relevant to your study. If you are not sure if a list item applies to your research, read the appropriate section before selecting a response.

Materials & experimental systems

n/a	Involved in the study
☒	☐ Antibodies
☒	☐ Eukaryotic cell lines
☒	☐ Palaeontology and archaeology
☒	☐ Animals and other organisms
☒	☐ Clinical data
☒	☐ Dual use research of concern
☒	☐ Plants

Methods

n/a	Involved in the study
☒	☐ ChIP-seq
☒	☐ Flow cytometry
☒	☐ MRI-based neuroimaging

Plants

Seed stocks	N/A
Novel plant genotypes	N/A
Authentication	N/A
